# Augmenting Basin-Hopping With Techniques From Unsupervised Machine Learning: Applications in Spectroscopy and Ion Mobility

**DOI:** 10.3389/fchem.2019.00519

**Published:** 2019-08-07

**Authors:** Ce Zhou, Christian Ieritano, William Scott Hopkins

**Affiliations:** Department of Chemistry, University of Waterloo, Waterloo, ON, Canada

**Keywords:** serine dimer, polyalanine, collision cross section, IRMPD, hierarchical clustering, potential energy surface, global optimization, vibrational spectroscopy

## Abstract

Evolutionary algorithms such as the basin-hopping (BH) algorithm have proven to be useful for difficult non-linear optimization problems with multiple modalities and variables. Applications of these algorithms range from characterization of molecular states in statistical physics and molecular biology to geometric packing problems. A key feature of BH is the fact that one can generate a coarse-grained mapping of a potential energy surface (PES) in terms of local minima. These results can then be utilized to gain insights into molecular dynamics and thermodynamic properties. Here we describe how one can employ concepts from unsupervised machine learning to augment BH PES searches to more efficiently identify local minima and the transition states connecting them. Specifically, we introduce the concepts of similarity indices, hierarchical clustering, and multidimensional scaling to the BH methodology. These same machine learning techniques can be used as tools for interpreting and rationalizing experimental results from spectroscopic and ion mobility investigations (e.g., spectral assignment, dynamic collision cross sections). We exemplify this in two case studies: (1) assigning the infrared multiple photon dissociation spectrum of the protonated serine dimer and (2) determining the temperature-dependent collision cross-section of protonated alanine tripeptide.

## Introduction

**Figure 1:**
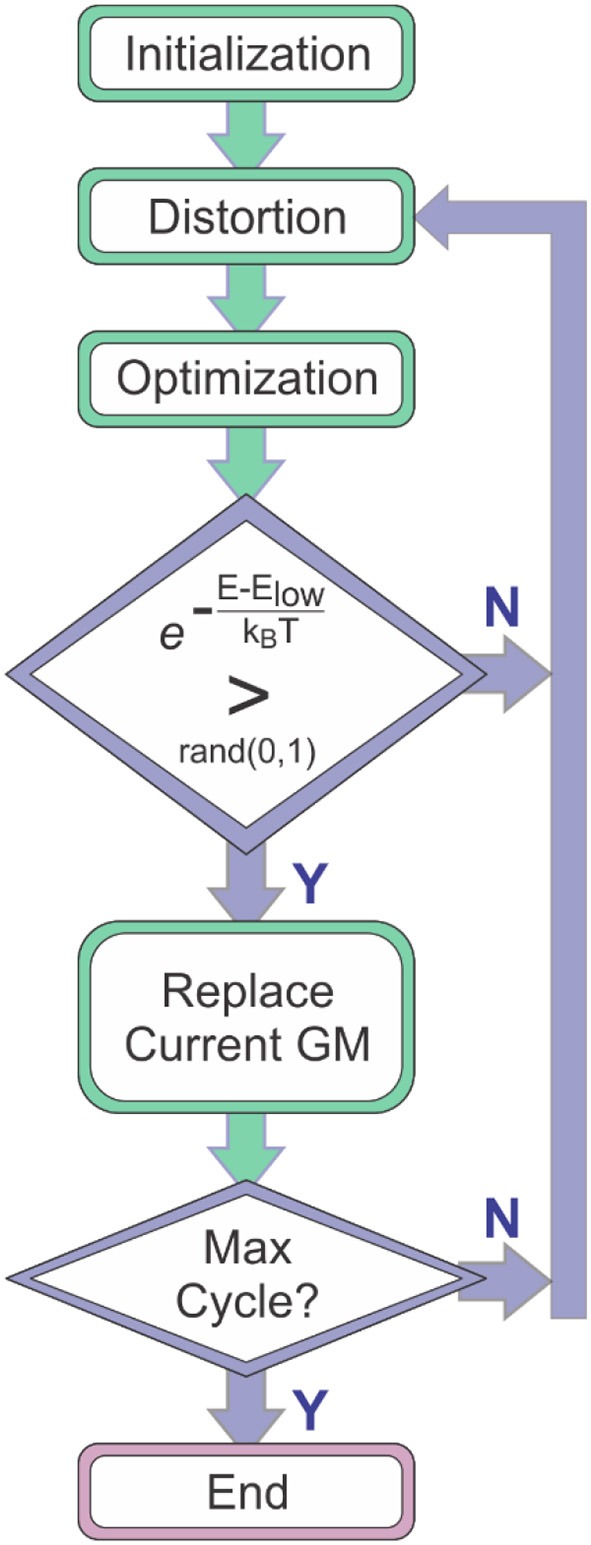
The general procedure of the basin-hopping algorithm. E_*low*_ is the energy of the lowest energy species identified to that point in the search (i.e., the current global minimum, GM).

Molecular global optimization (GO) to identify the chemically-relevant species on hypergeometric potential energy surfaces (PESs) provides both rationalizations and predictions of experimental observations by relating thermodynamic and kinetic properties to the accessible local minima and the transition states (TSs) that connect them (Scheraga, 1992; Piela et al., 1994; Wales and Doye, 1997; Wales and Scheraga, 1999). Basin-hopping (BH) is a technique for GO that is based on the iterative approach of performing random perturbation of geometric coordinates, local optimization of a model potential energy function, and accepting or rejecting the perturbed coordinates based on the value of the minimized function (Wales and Doye, 1997; Wales et al., 1998; Wales and Scheraga, 1999; Lecours et al., 2014). Use of the BH algorithm for searching molecular PESs was outlined by Wales and Doye in their 1997 article “Global Optimization by Basin-Hopping and the Lowest Energy Structures of Lennard-Jones Clusters Containing up to 110 Atoms,” (Wales and Doye, 1997) which describes how the technique transforms the PES into a collection of interpenetrating staircases wherein each stair/plateau on the transformed surface is associated with a stationary point (usually local minimum) of the original potential energy landscape. Figure 1 shows a flow diagram outlining the general procedure of the BH search algorithm. The key feature of the BH algorithm is the inclusion of assessment criteria for accepting or rejecting a newly distorted input geometry. One of these criteria is the definite replacement of the lowest energy structure identified by the BH routine with the currently optimized structure if that structure has a lower energy. A second key criterion is a conditional acceptance of the distorted geometry by assessing the statistical accessibility of the optimized structure based on a pre-defined energy window. For example, one can define a Boltzmann distribution at a given temperature with respect to the current lowest energy structure and assess the probability of accessing the newly generated stationary point. Thus, the BH algorithm has a bias toward low energy structures and is a good option for identifying the global minimum (GM) and local minima that may be present in an ensemble under thermal equilibrium conditions.

To further improve the efficiency of a BH search, one can include additional criteria for assessment of distorted molecular geometries prior to optimization. For example, one might choose to reject structures in which inter-atomic distances are less than some pre-defined threshold, or one might choose to define an interaction volume to prevent molecular/cluster dissociation (Lecours et al., 2014). It is also common to select specific degrees of freedom (DoFs) for random distortion while freezing others; one might choose to search the conformational space defined by molecular dihedral angles while leaving the distances between chemically bonded atoms fixed (Hopkins et al., 2013, 2015). There are several other works which employ more dramatic changes to the underlying BH algorithm. For example, Leary proposed a version in which only the replacement criterion is employed in the evaluation (i.e., no statistically accessible energy window is specified) (Leary, 2000). In other works, Röder and Wales propose a mutational BH algorithm to optimize biomolecules (Röder and Wales, 2018), and Kim et al. combine BH with Coulomb matrix analysis to sample reaction intermediates (Kim et al., 2014). While these variants have all been successful in the task at hand, the fact that the basic BH algorithm often requires tailoring highlights the inherent drawbacks in the BH methodology.

One principal short-coming of the BH algorithm that practitioners must be aware of is that the method is not deterministic; i.e., identifying the GM via a finite, stochastic search is not guaranteed. Confidence in BH search results come from a satisfactory agreement with experimental observations and/or the consistency of results from several parallel simulations with different initial conditions. A second potential short-coming is the fact that, due to performance considerations, BH calculations are often conducted with relatively low-level model chemistries (e.g., molecular mechanics), which may not be accurate enough for certain molecular systems. Finally, practitioners must be aware that a BH search may be kinetically trapped in a local potential minimum if the thermal energy (*viz*. temperature) of the simulation is set too low. In fact, in some cases BH searches of PESs are non-ergodic regardless of simulation temperature. For example, consider the case of protonated *para*-aminobenzoic acid, which can exhibit protonation on either the carbonyl oxygen atom or the amine nitrogen atom in the gas phase (Tian and Kass, 2009; Schmidt et al., 2011; Campbell et al., 2012, 2016). If one were to assume that the protonation site of *para*-aminobenzoic acid were the nitrogen center (as is the case in protic solution) and model the system as a molecular cation using a molecular mechanics force field, the O-protonated isomer (which is the gas phase global minimum) would not be identified without modifying the atomic connectivity during the BH search (Tian and Kass, 2008; Campbell et al., 2012, 2016). To overcome this systematic limitation, one must treat the charge-carrying proton as a separate moiety in the simulation and/or augment the BH framework with the chemical intuition of the user (i.e., manually identify both prototropic isomers and conduct BH searches for each of them).

Here, we describe how the basin-hopping algorithm can be employed to reliably model gas phase cluster and molecular systems for comparison with observations from spectroscopy and ion mobility experiments. To model our experimental observations, we require theoretical predictions from a collection of local minima, which do not necessarily include the global minimum, and an efficient method to find matches between the predictions and the observations. In some cases, it is also desirable to identify the TSs that connect minima to assess thermodynamic accessibility of the various isomers / conformers. These two requirements present two notable challenges for the BH methodology. The first challenge, related to the principal short-coming mentioned above, is the necessity to accurately track the explored regions of the PES. In doing so, one not only identifies a set of local minima, but also gains useful information for directing the BH search toward regions of the PES that are relatively unexplored. The second challenge is the accurate and efficient identification of the TSs that connect local minima. To overcome these challenges, we collect the nuclear configuration data that is generated during the BH search and utilize this data as described in Section Augmenting the BH Algorithm. Specifically, in Section Assessing Geometric Similarity we describe how one can utilize similarity functions and hierarchical clustering, which are concepts generally associated with unsupervised machine learning, to assess the uniqueness of the local minima and guide PES searches. We then discuss the interpolation of geometries to identify intermediate local minima and to create guess geometries for TS searches in Section Interpolating Intermediate Geometries. In Section Application of BH Search Results, we outline our methods for employing our BH results to assign the spectral carriers (Section Case Study 1: The IR Spectrum of the Protonated Serine Dimer) and to model temperature-dependent structures (Section Case Study 2: Dynamic Collision Cross Section of Protonated Alanine Tripeptide) of geometrically-fluxional species. Finally, we summarize our perspective and highlight open questions in Section Conclusions.

## Augmenting the BH Algorithm

As mentioned in section Introduction, several variations to the BH algorithm have been proposed to address specific challenges in searching complex potential energy landscapes (Leary, 2000; Kim et al., 2014; Röder and Wales, 2018). For our purposes, where it is necessary to identify a collection of local minima that are representative of the species present in experimental ensembles, we require a faithful mapping of the molecular PES. To improve the efficiency and PES coverage of the BH algorithm, we introduce a method of comparing the geometries of local minima. This comparison, which is derived from a similarity function, provides a more rigorous identification of unique isomeric species and insight into which regions of the PES may require additional exploration.

In analogy to the spatial distance between two locations on a map, a similarity function quantifies the similarity of two conformations, A and B, in conformation space. The function, usually denoted as *d*(A,B), is non-negative (*d*(*A, B*) ≥ 0), symmetric (*d*(*A, B*) = *d*(*B, A*)) and has zero value only when two identical elements are evaluated (*d*(*A, A*) = 0) (Locatelli and Schoen, 2013). The similarity function can be used in one of three ways: qualification, quantification, and interpolation. Qualification usage implies that the function need only tell if two input structures are identical. Quantification usage provides a metric for how much difference is there between two structures; for example, is structure A more similar to structure B than to structure C? Interpolation usage means that, given two structures, A and B, and an arbitrary interpolation factor, λ ∈ (0, 1), there exist one or more structures, C, satisfying:

(1) $$\begin{array}{r} d\left(A,B\right)=\frac{1}{\lambda }d\left(A,C\right)=\frac{1}{1-\lambda }d\left(B,C\right) \end{array}$$

If the function *d* satisfies triangular inequality *d*(*A, B*) + *d*(*B, C*) ≥ *d*(*A, C*)), the structure C is unique, and *d* is a metric of the conformation space (Choudhary, 2003). Note that special treatment is required if A and B have different numbers of atoms (i.e., if A and B are of different dimension); this tends not to be the case in simulations of chemical systems. The interpolation mechanism is of central importance not only to a number of GO algorithms, such as particle swarm optimization (Eberhart and Yuhui, 2001), differential evolution (Storn and Price, 1997), and DIRECT (Jones et al., 1993), but also to unsupervised machine learning techniques such as the self-organizing map (Kohonen, 1990) and the growing neural gas (Martinez and Schulten, 1991; Fritzke, 1994). In qualitative comparisons, the similarity function need only account for the translational, rotational, and permutational invariance under a given molecular representation; structural equivalence only occurs between species of identical composition. Such invariance properties are either embedded in the mathematical definition of the molecular representation or they are achieved via manually aligning the two molecular systems prior to evaluating their similarity. Examples of such representations include the conventional skeletal chemical formula and the SMILEs code used in compound database systems (Weininger, 1988; Rahman et al., 2009; Heller et al., 2013). In quantitative comparisons, the similarity of two structures is specified by a real number. These similarity indices are useful in discriminating visited regions of the PES (e.g., well-sampled vs. poorly-sampled regions), which can be assessed using unsupervised machine learning analyses like hierarchical clustering and multidimensional scaling (MDS) (Wickelmaier, 2003; Borg and Groenen, 2005). Most similarity functions used for quantitation purposes are defined by the normal (e.g., the root-mean-square deviation of atomic positions, RMSD) (Kabsch, 1976) or reciprocal (e.g., the Coulomb matrix) (Montavon et al., 2012) interatomic distances, although electron density-based similarity functions have found use in drug discovery (Cereto, 2015; Kumar and Zhang, 2018). To implement structural interpolation, the back conversion from desired similarity constraints to a concrete structure is required. This technique enables generation of intermediate geometries for TS calculations (e.g., QST3) (Peng and Bernhard Schlegel, 1993; Peng et al., 1996), and it can also be used to guide BH searches of specified regions of the PES along isomerization pathways between two isomers. Furthermore, by implementing structural interpolation, one creates the opportunity to incorporate other GO techniques (e.g., particle swarm optimization) (Kennedy and Eberhart, 1995; Call et al., 2007; Shi et al., 2019) and machine learning techniques (e.g., growing neural gas) (Martinez and Schulten, 1991; Fritzke, 1994) into the BH algorithm. In practice, rather than an explicit analytical approach, structure interpolation can be achieved implicitly via local optimizations with a tolerable loss of accuracy. In our research, to efficiently use the nuclear configuration information from the BH simulation, we introduce both Euclidean distance matrix-based and cosine distance-based similarity functions together with the necessary techniques to accomplish structural interpolation. The mathematical and implementation details are described below.

### Assessing Geometric Similarity

To begin assessing the similarity between two molecular geometries, one must first select an appropriate similarity function. One option, the Euclidean distance matrix representation (***D***) of a molecule, is simply the collection of all interatomic distances as per (Gentle, 2007):

(2) $$\begin{array}{r} D_{ij}=|{\vec{r}}_{i}-\;{\vec{r}}_{j}| \end{array}$$

where ${\vec{r}}_{i}$ and ${\vec{r}}_{j}$ are the positional vectors (in Cartesian coordinates) of atoms *i* and *j*. Within the distance matrix representation, the similarity function is defined as the sum of the absolute difference between each atom pair for structures A and B:

(3) $$\begin{array}{r} d\left(D_{A},D_{B}\right)=\sum i,j>i|D_{A,ij}-D_{B,ij}| \end{array}$$

The distance matrix is a symmetric matrix with diagonal elements of zero. This representation is translationally and rotationally invariant, but not permutationally invariant (*viz*. identical nuclei are not necessarily chemically equivalent). Thus, in practice, the atom labeling should be adjusted such that the similarity index (the value of the similarity function) of the two input molecules is minimized. It should be noted that the memory requirement of this representation scales quadratically with the number of atoms. Consequently, the distance matrix approach is not a good choice for dealing with very large systems.

A second option is to represent the molecular nuclear configuration as a vector, *R*^⇀^ (Fu and Hopkins, 2018), containing the mass-weighted distance between each atom and the molecular center-of-mass:

(4) $$\begin{array}{r} {\vec{R}}_{COM}=\frac{\sum_{i}^{m_{i}}{\vec{r}}_{i}}{\sum_{i}m_{i}} \end{array}$$

(5) $$\begin{array}{r} {\vec{R}}_{i}=m_{i}\left|{\vec{r}}_{i}-{\vec{R}}_{\text{COM}}\right| \end{array}$$

Where *m*_*i*_ and ${\vec{r}}_{i}$ are the mass and the distance to the center-of-mass for the *i*^th^ atom. Given that the mass-weighted distance vector representation is in the center-of-mass frame, one can then calculate the cosine distance between the vectors for isomers A and B as per:

(6) $$\begin{array}{r} d({\vec{R}}_{A},{\vec{R}}_{B})\;=\;\frac{\cos^{-1}\left(s({\vec{R}}_{A},{\vec{R}}_{B})\right)}{\pi } \end{array}$$

Where

(7) $$\begin{array}{r} s({\vec{R}}_{A},{\vec{R}}_{B})=\frac{{\vec{R}}_{A}{\;}^{\cdot }{\vec{R}}_{B}}{\left|\;{\vec{R}}_{A}\right|\left|\;{\vec{R}}_{B}\right|} \end{array}$$

Again, this representation is translationally and rotationally invariant. However, care should be taken to ensure that the identity of the *i*th atom is retained throughout the BH search so that one compares the same atoms in each unique geometric structure. Alternatively, one might choose an operational convention whereby the resulting vector is sorted (e.g., smallest to largest values) prior to calculating cosine distance; this introduces a permutational invariance to the treatment for low symmetry systems. In contrast to the quadratic scaling of the distance matrix, the mass-weighted distance vector scales linearly with number of atoms. However, as a trade-off, the mass-weighted distance vector representation is less effective than the distance matrix approach in discriminating between conformers of highly symmetric species. For example, the mass-weighted distance vector representation is unable to distinguish square planar and tetrahedral conformations of methane given identical C–H bond length. Nevertheless, the uniqueness of the isomer-vector correspondence is still largely guaranteed in most cases in which only low symmetry structures are considered, particularly when relative energies are also considered in distinguishing isomeric/conformeric species.

**Figure 2:**
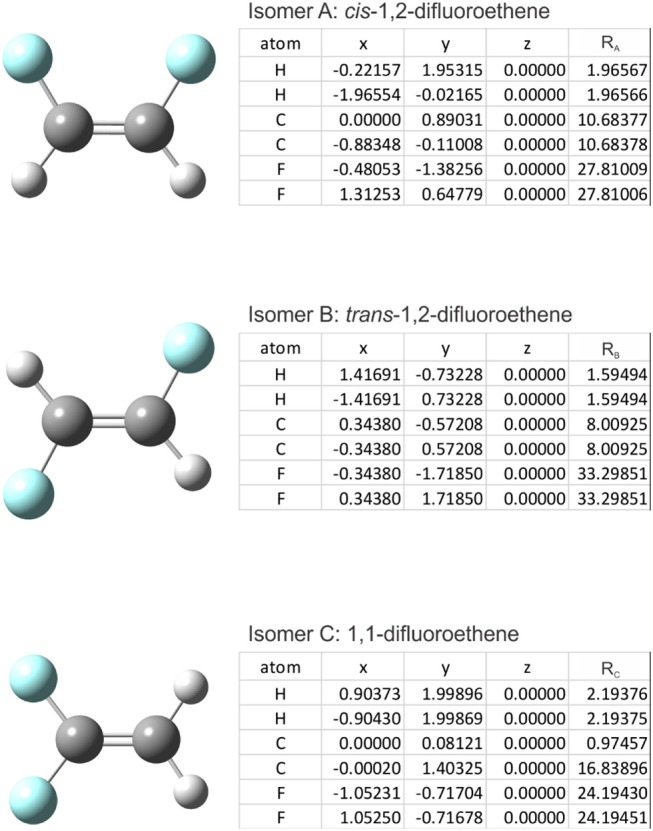
The structures of **(top)** *cis*-1,2-difluoroethene, **(middle)** *trans*-1,2-difluoroethene, and **(bottom)** 1,1-difluoroethene. **(Inset Tables)** atomic coordinates and the mass-weighted distance vectors. Geometries were optimized at the PM6 level of theory as implemented in Gaussian 16 (Frisch et al., 2016).

The cosine similarity (Equation 7) ranges from −1 (meaning exactly opposite) to +1 (meaning identical). However, in practice, the cosine similarity for real molecular structures ranges from 0 to 1 since the center-of-mass vector is constructed from real space distances, which are always positive. Thus, two identical structures exhibit mass-weighted distance vectors with zero angular distance between them, and angular distances between vectors increase as the differences between the geometric structures of the associated isomers increase. For example, consider the isomers *cis*-1,2-difluoroethene, *trans*-1,2-difluoroethene, and 1,1-difluoroethene shown below in Figure 2. By inspection, one can identify that the mass-weighted distance vectors for the *cis*-1,2-difluoroethene and *trans*-1,2-difluoroethene isomers (R_A_, R_B_) are more like one another than they are to that of the 1,1-difluoroethene isomer (R_C_). This is confirmed when calculating the cosine distances (see Table 1).

**Table 1:** The cosine distance matrix for *cis*-1,2-difluoroethene, *trans*-1,2-difluoroethene, and 1,1-difluoroethene.

**Distance**	***cis*-1,2-difluoro**	***trans*-1,2-difluoro**	**1,1-difluoro**
*cis*-1,2-difluoro	0	0.04200	0.09497
*trans*-1,2-difluoro	0.04200	0	0.10219
1,1-difluoro	0.09497	0.10219	0

*Geometries were optimized at the PM6 level of theory as implemented in Gaussian 16 (Frisch et al., 2016)*.

Calculating the distances between molecular structures facilitates analysis through agglomerative hierarchical clustering (Day and Edelsbrunner, 1984). This analysis provides a visual representation of the similarity of geometric structures—via production of a dendrogram plot—and therefore provides some insight into which species occupy similar regions of the potential energy landscape with respect to the mass-weighted nuclear coordinates. There are several methods available for analysis via agglomerative hierarchical clustering (Day and Edelsbrunner, 1984). One option for this analysis is the weighted pair group method with arithmetic mean (WPGMA), developed by Sokal and Michener (Michener and Sokal, 1957; Sokal and Michener, 1958). In each iteration of the WPGMA algorithm, the two nearest species (P and Q) are combined into a higher-level group P ∪ Q, thereby reducing the dimension of the *m* × *m* distance matrix (e.g., Table 1) by one row and one column. The distance between group P ∪ Q and another group R is the arithmetic mean of the distances between the members of P ∪ Q and R, i.e.,:

(8) $$\begin{array}{r} d_{\left(P\cup Q\right),R}=\frac{d_{P,R}+d_{Q,R}}{2} \end{array}$$

**Figure 3:**
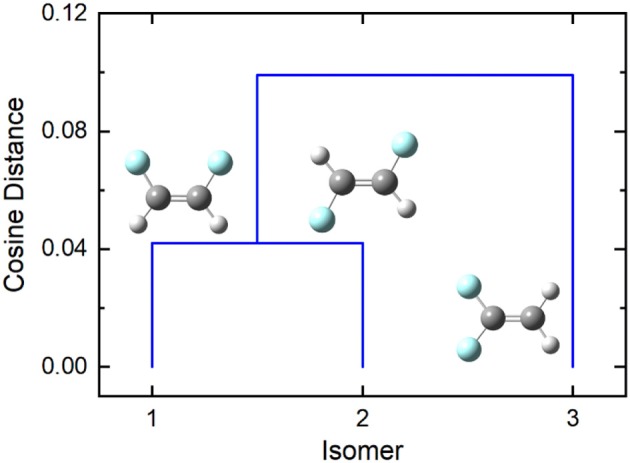
The cosine distance dendrogram for difluoroethene. Molecular geometries were optimized at the PM6 level of theory as implemented in Gaussian 16 (Frisch et al., 2016).

In the case of difluoroethene (Figure 2 and Table 1), the smallest cosine distance of 0.042 between the *cis-* and *trans-*1,2-difluoroethene isomers would lead to their clustering as P ∪ Q, and the distance between this higher-level group and the 1,1-difluoroethene isomer would be (0.09497 + 0.10219)/2 = 0.09858. A dendrogram showing the hierarchical clustering of the isomers of difluoroethene is provided in Figure 3. By inspection of the dendrogram one can immediately see that the *cis-* and *trans-* isomers of 1,2-difluoroethene isomers are more closely related geometrically than either of these isomers is related to 1,1-difluoroethene.

### Interpolating Intermediate Geometries

When searching complex PESs to find local minima or TSs, it is sometimes useful to interpolate geometries that are intermediate to two previously identified isomers. For example, consider the case in which a set of isomeric species has been identified, but one is very dissimilar from the others as determined by the geometric analysis described above. This might indicate that the BH search has become kinetically trapped and more attention should be paid to the region of the PES associated with the isolated structure. It is then useful to explore the PES between the more extensively mapped region and the region associated with the isolated structure to search for intermediates along the isomerization pathway and/or identify barriers to isomer interconversion. For the purpose of generating initial guess structures for the BH algorithm or for QST3 TS calculations, precise interpolation is not always necessary; (Peng and Bernhard Schlegel, 1993; Peng et al., 1996) most of the time interpolation can be accomplished implicitly, thereby improving the efficiency of the PES mapping. Currently, we have implemented two classes of implicit interpolation methods, one based on Monte Carlo sampling and the other based on molecular dynamics simulation.

Since the acceptance criteria are replaceable as a standard module in the evaluation part of the BH framework, instead of searching for low energy structures, one can choose to sample structures between two given minima on the PES within specified similarity constrains. Thus, a Monte Carlo with minimization approach can be established along a specified path/region of the PES. By applying an upper threshold to the distance of the sampled structure from the minima, one can constrain the search to a hyperdimensional ellipsoidal space between the two minima of interest. Within the distance matrix representation, the interpolation can also be accomplished with optimization on an interpolated artificial force field. Similar to the idea of the artificial force induced reaction (Maeda et al., 2014), the interpolated structure is obtained by minimizing a molecular mechanics-type force field, ***V***:

(9) $$\begin{array}{r} V(D_{C})=\frac{\chi }{{\bar{r}}_{ij}}{\left(D_{C,ij}-{\bar{r}}_{ij}\right)}^{2} \end{array}$$

where χ is an arbitrary constant that facilitates optimization, and *D*_*C, ij*_ and ${\bar{r}}_{ij}$, are the actual and expected interatomic distance of the interpolated structure. ${\bar{r}}_{ij}$ is constructed from the two minima, D_A_ and D_B_ and the interpolation factor, λ (0 ≤ λ ≤ 1) as per:

(10) $$\begin{array}{r} {\bar{r}}_{ij}=\lambda D_{A,ij}+(1-\lambda )D_{B,ij} \end{array}$$

The force field is thus a collection of harmonic terms whose force constant is inversely proportional to ${\bar{r}}_{ij}$. Compared to the Monte Carlo approach, using this force field approach in conjunction with standard geometry optimization techniques is expected to be more efficient at identifying intermediate structures owing to the reduced and more pertinent search space.

## Application of BH Search Results

Experimental measurements are typically concerned with probing ensembles, rather than single molecules. Consequently, it is necessary to identify which structures are present in the probed ensemble and the relative populations of those species. This can be particularly challenging for chemical systems that are kinetically trapped in a relatively high-energy region of the PES and for systems that are fluxional (i.e., those that can easily access multiple minima on the experimental time scale). To demonstrate the potential of our augmentation to the original BH method, we describe our efforts to model the infrared multiple photon dissociation (IRMPD) spectrum of proton-bound serine dimer and the temperature-depending collision cross section (CCS) of protonated alanine tripeptide, [AAA+H]^+^.

### Case Study 1: The IR Spectrum of the Protonated Serine Dimer

IRMPD spectroscopy has become one of the most effective techniques for determining the structure of molecular ions (Jašíková and Roithová, 2018). Ion spectra are recorded by isolating a specified *m/z* species in an ion trap and monitoring the fragmentation efficiency of the molecular ion as a function of the frequency of a probe laser, which passes through the ion trap, intersecting with the ion cloud (Lemaire et al., 2002; Oh et al., 2005; Polfer, 2011). Thus, IRMPD spectroscopy is a type of action spectroscopy whereby molecular fragmentation is interpreted as a signature of photon absorption. A detailed description of the technique is available in references (Aleese et al., 2006) and (Macaleese and Maître, 2007). By probing in the IR region, one obtains information on the frequencies of fundamental vibrational transitions, which may then be compared with the harmonic (and sometimes anharmonically-corrected) vibrational frequency predictions of electronic structure software packages. This, in turn, facilitates structural assignment based on the similarity between computed and measured spectra, and the identification of distinguishing/diagnostic spectral features.

Spectroscopic investigation of amino acids and amino acid-containing clusters continues to be an active field of research owing to the biological relevance of these systems (Nanita and Cooks, 2006; Mino et al., 2011; Stedwell et al., 2013; Sunahori et al., 2013; Armentrout et al., 2014; Seo et al., 2017, 2018; Heiles et al., 2018; Jašíková and Roithová, 2018; Ma et al., 2018; Scutelnic et al., 2018). In particular, serine has received a great deal of attention owing to the implication of the serine octamer in homochiral genesis (i.e., the origin of L-amino acid chiral preference in nature) (Counterman and Clemmer, 2001; Sunahori et al., 2013; Seo et al., 2017; Scutelnic et al., 2018). Indeed, the Bowers and von Helden groups recently published a series of high-profile studies detailing the assignment of the IR spectra for cryogenically-cooled protonated serine octamer, [Ser_8_ + H]^+^, and protonated serine dimer, [Ser_2_ + H]^+^ (Seo et al., 2017, 2018; Scutelnic et al., 2018). To demonstrate the utility of our augmented BH approach for searching PESs and assigning IR spectra, we employed our methodology to study [Ser_2_ + H]^+^.

To begin, preliminary B3LYP/6-311++G(d,p) optimizations were conducted for neutral and protonated serine monomers to obtain partial charges for utilization with the molecular mechanics force field. For neutral monomers, both canonical and zwitterionic initial guesses were employed, and only the canonical structures were obtained. For the protonated isomers, initial guesses protonated at the carbonyl group, the amine group, and the side-chain hydroxyl group were optimized; all resulted in an amine-protonated structure, in agreement with previously published results (Noguera et al., 2001). After the optimizations, the atomic partial charges were calculated using the CHelpG partition scheme to reproduce the electrostatic potential at the near exterior of the van der Waals radial surface (Breneman and Wiberg, 1990). DFT optimizations were run in parallel, threaded across 8 cores, and required approximately 1 hour per calculation. Following pre-optimization and partial charge calculations for the monomers, both moieties were combined to produce the protonated dimer for treatment with the BH code. To search the potential energy landscape, dihedral angles in both moieties were given random rotations of −5° ≤ ϕ ≤ +5° on each iteration of the BH algorithm. The neutral moiety was also given random rotations of −5° ≤ θ ≤ +5° around its body-fixed *x*–, *y*–, and *z* –axes, and random translation of −0.5 Å ≤ η ≤ +0.5 Å in each of the *x*–, *y*–, and *z*–directions. This ensures that the relative orientations of the two moieties are also sampled. For geometry optimization, the custom-written BH code interfaces with the Gaussian software package where the AMBER force-field is used as the model potential (Wang et al., 2006; Frisch et al., 2009). Following an initial run of 1,000 steps at a thermal energy of E ≈ 0.43 eV (T = 5,000 K) to generate candidate structures, several parallel BH runs of 10,000 steps were run at a thermal energy of E ≈ 0.09 eV (T = 1,000 K) to search the PES. In total, more than 60,000 cluster geometries were sampled.

To benchmark the augmented BH algorithm, eight standard BH simulations of 5,000 steps were conducted and structural interpolation was subsequently applied to the unique isomers identified at the PM7 level of theory. Unique [Ser_2_ + H]^+^ isomers were identified based on energetic differences (ΔE ≥ 10^−5^ Hartree) and by using a value of 50.0 Å as the similarity threshold between isomer pairs within the Euclidean distance matrix (*vide supra*). Isomer pairs with Euclidean distances of more than 150.0 Å were candidates for structural interpolation. Due to the large number of potential isomer pairs (~6,000 for each BH simulation), we chose to randomly select only 300 pairs to test the interpolation methodology. For each pair, the midpoint structure (λ = 0.5) was located as described above and optimized at the PM7 level of theory. The optimized geometry of the interpolated structure was then compared to those in the original BH set using the same energy and Euclidean distance thresholds as employed previously. The results of the eight parallel (BH + interpolation) simulations are summarized in Table 2.

**Table 2:** The results of eight (BH + interpolation) simulations of [Ser_2_ + H]^+^.

**Simulation**	**# Isomers found**		**Global minimum (Hartree)**	
	**BH**	**+ Interpolation**	**BH**	**Interpolation**
1	70	16	**−0.25984**	−0.25940
2	74	19	**−0.25984**	−0.25593
3	60	8	**−0.25984**	−0.25572
4	67	22	−0.25969	**−0.25984**
5	62	32	−0.25969	**−0.25984**
6	76	17	**−0.25984**	−0.25967
7	67	6	**−0.25984**	−0.25515
8	51	28	−0.25969	−0.25809

*Geometries were optimized at the PM7 level of theory (Frisch et al., 2016). Bold values emphasize the lowest energy value among all the isomers obtained from the total of 8 searches*.

There are two observations worth noting in Table 2. Firstly, the isomer sets that were identified by the standard BH algorithm are augmented considerably by post-simulation interpolation; on average 19 new isomers were identified by interpolating between the 300 randomly selected isomer pairs found by standard BH simulations. Secondly, although the global minimum structure was identified in only five of the eight standard BH simulations of 5,000 steps, introducing post-simulation interpolation improved the rate of identifying the [Ser_2_ + H]^+^ global minimum to seven out of eight simulations.

**Figure 4:**
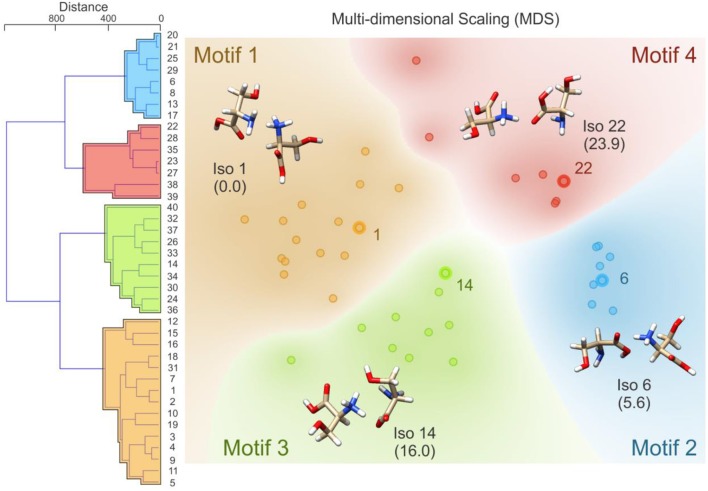
**(Left)** The distance dendrogram for the protonated serine dimer. Isomer numbers are indicated for each branch of the dendrogram. **(Right)** A multi-dimensional scaling 2D projection of the hierarchical clustered data. Isomers are numbered in order of increasing energy above the global minimum (isomer 1). Standard Gibbs energies (in parentheses) are reported in kJ mol^−1^. Calculations were conducted at the B3LYP/6-311++G(d,p) + GD3 level of theory as implemented in Gaussian 09 (Frisch et al., 2009).

Following BH simulation, the 200 unique lowest energy structures were carried forward to re-optimization at the B3LYP/6-311++G(d,p) + GD3 level of theory (Becke, 1988, 1993; Grimme et al., 2010). This treatment reduced the total number of unique isomers to 40. To ensure that these structures were local minima on the PES (i.e., no negative eigenvalues in the Hessian matrix, rather than TSs which have one negative Hessian eigenvalue), harmonic frequency calculations were undertaken. These calculations also served to predict the vibrational (*viz*. IR) spectra of the isomers and to estimate thermochemical corrections (see sections 1.1 and 1.2 of the Supplementary Materials for details). Using the optimized geometries from the density functional theory calculations, the distance matrix (as described in Equations 2, 3) was constructed. Linkages for hierarchical clustering were then determined using Ward's minimum variance method as implemented in the Orange software package (https://orange.biolab.si/) (Demsar et al., 2013), which at each step finds the pair of clusters that leads to the minimum increase in total within-cluster variance after merging (Ward, 1963). The resulting dendrogram, which is plotted in Figure 4, clearly shows four distinct groups of geometric structures; these groups are highlighted in blue, red, green, and orange. To better visualize the data, we have also used multi-dimensional scaling to create a 2D plot of the clustered data (Wickelmaier, 2003; Borg and Groenen, 2005). Based on this hierarchical clustering analysis, we clearly see that the BH algorithm identified several local minima associated with four distinct regions of the [Ser_2_ + H]^+^ PES. The lowest energy isomer in each of these four regions (*viz*. isomers 1, 6, 14, and 22) are highlighted and labeled on the MDS plot. This type of analysis provides insight with respect to how thoroughly a region of the PES has been searched. For example, if only one or two data points were identified in the blue region of the MDS plot, one might decide to initialize an additional BH run starting from one of the previously identified geometries. Moreover, this analysis can help guide interpolation efforts to identify TSs or geometries associated with stable intermediates between two previously identified minima. For example, upon inspection of the MDS plot shown in Figure 4, one can identify two outliers associated with the red group (in the top left of the red section) and one outlier associated with the green group (bottom left of the green section). In principle, one might choose to explore the region between these features and the more closely clustered structures on the MDS plot via the methods described in section Interpolating Intermediate Geometries. We choose not to do so here, however, because these three structures are associated with isomers 38, 39, and 40 (the highest energy species in our set).

**Figure 5:**
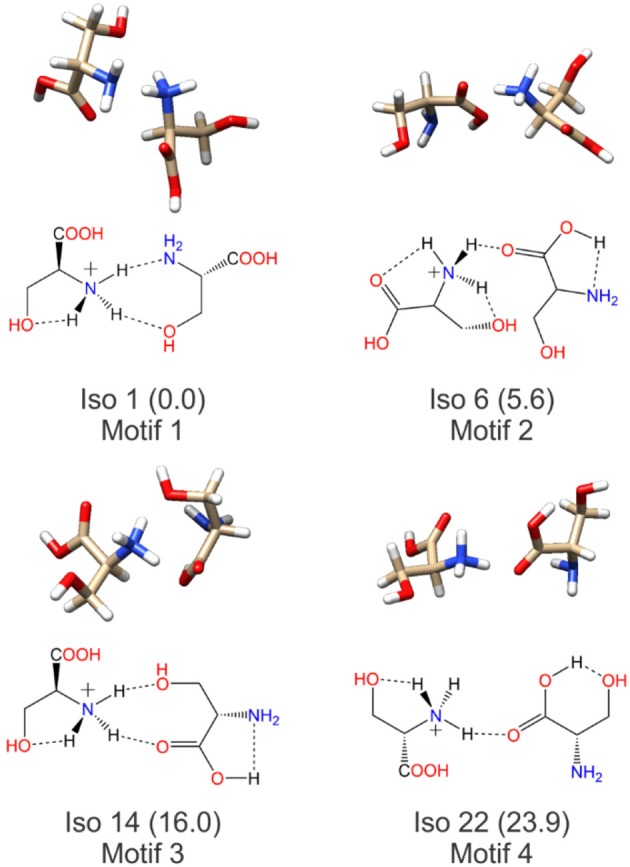
The lowest energy isomers for each low energy binding motif of the protonated serine dimer. Motifs 1 and 3 show bidentate coordination between the two moieties, whereas motifs 2 and 4 exhibit monodentate coordination between the two moieties.

Having identified four low energy geometric groupings associated with the [Ser_2_ + H]^+^ PES, we can then visually inspect the structures to rationalize their association via hierarchical clustering. In doing so, we find that the clustered species are associated with four distinct binding motifs, which we label motifs 1 (orange), 2 (blue), 3 (green), and 4 (red). The 3D structures and 2D chemical structures for the lowest energy isomer in each group is provided in Figure 5. Motifs 1 and 3 are associated with bidentate complexation between the ammonium group of the protonated moiety and the neutral moiety. In the case of motif 1, the ammonium group forms intermolecular hydrogen bonds with the amino group and the hydroxyl group of the neutral moiety. In contrast, motif 3 forms intermolecular hydrogen bonds with the hydroxyl group and the carboxylic acid group of the neutral moiety. Motifs 2 and 4 are associated with monodentate complexation between the ammonium group of the protonated moiety and the neutral moiety. These two binding motifs differ in terms of the relative orientations of the two serine moieties and with respect to the presence of a O–H•••N intramolecular hydrogen bond (IMHB) in the neutral moiety (motif 2) versus a O–H•••O IMHB in the neutral moiety (motif 4).

To determine which (if any) of the computed [Ser_2_ + H]^+^ isomers are observed experimentally, calculated harmonic vibrational spectra were compared against the experimental IRMPD spectrum using the methodology outlined by Fu and Hopkins (2018) The experimental spectrum employed was a concatenation of the spectra recorded by Seo et al. in the 1,000–1,900 cm^−1^ region and by Sunahori et al. in the 3,200–3,800 cm^−1^ region (Sunahori et al., 2013; Seo et al., 2018). These spectra were digitized using a custom-written python script from figures in their respective publications, interpolated in 2 cm^−1^ intervals, then normalized such that the maximum intensity in each region was set to 1. Calculated IR spectra were first scaled using appropriate frequency scaling factors and broadened with a Lorentzian line shape of 15 cm^−1^ FWHM (Andersson and Uvdal, 2005; Fu and Hopkins, 2018), and then were similarly interpolated and normalized. The intensity vectors (i.e., y-values) of the computed spectra were then compared with the experimental spectrum by taking the Euclidian distance (*d*_*Euc*_) between the intensity vectors and assigning a scaled similarity index as per:

(11) $$\begin{array}{r} Scaled\;Similarity=1-\frac{\left(d_{Euc}-d_{Euc}^{Min}\right)}{\left(d_{Euc}^{Max}-d_{Euc}^{Min}\right)} \end{array}$$

**Figure 6:**
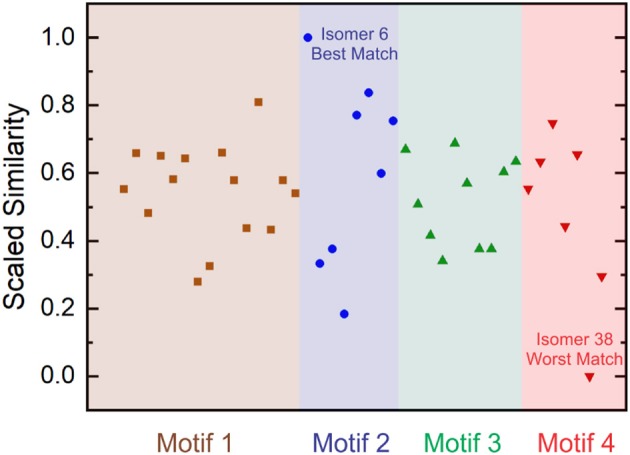
Scaled Euclidean similarities of computed harmonic vibrational spectra to experimental IRMPD spectra for the protonated serine dimer. Isomer 6 gives the best match and Isomer 38 gives the worst match amongst the 40-isomer set. Isomers are ordered in increasing energy from left to right in each motif.

Where $d_{Euc}^{Min}$ is the minimum Euclidean distance amongst the set of vectors and $d_{Euc}^{Max}$ is the maximum Euclidean distance amongst the set of vectors following subtraction of the minimum distance. This treatment generates a scaled similarity index that ranges between 0 (worst match) and 1 (best match). The scaled similarities for the computed [Ser_2_ + H]^+^ isomer spectra are plotted in Figure 6. Inspection of Figure 6 indicates that Isomer 6 yields a significantly better match to the experimental spectrum than do other isomers. Moreover, we find that four of the five best matches are provided by isomers associated with binding motif 2. This suggests that, despite the fact that motif 1 is associated with the lowest energy region of the [Ser_2_ + H]^+^ PES at T = 298 K and P = 1 atm, the region of the PES associated with motif 2 is predominantly populated in ion trap experiments.

**Figure 7:**
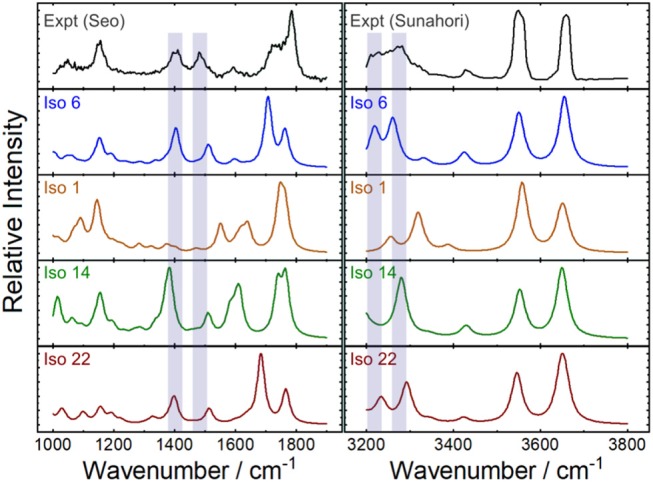
Experimental IRMPD spectra and computed harmonic vibrational spectra for the protonated serine dimer. The experimental spectra were adapted from Seo et al. (2018) and Sunahori et al. (2013). The computed IR spectra are associated with the lowest energy isomer for each of the four binding motifs. Scaling factors of 0.9679 and 0.95 were employed for the 1,000–1,900 cm^−1^ and 3,200–3,800 cm^−1^ regions, respectively (Andersson and Uvdal, 2005; Fu and Hopkins, 2018).

Figure 7 plots the experimental IRMPD spectrum for [Ser_2_ + H]^+^ and the computed spectra for isomers 1, 6 (best match), 14, and 22—the lowest energy isomers associated with each of the four binding motifs. The diagnostic peaks, which are highlighted in blue in Figure 7, are associated with the HNH angle bending motions (*ca*. 1,450 cm^−1^) and N–H bond stretching motions (*ca*. 3,250 cm^−1^) of the ammonium and amino groups. Although isomer 1 is the global minimum structure based on standard Gibbs energies, the spectrum of isomer 6 (+5.6 kJ mol^−1^) is much more representative of the experimental spectrum. This was also noted by Sunahori et al., who identified isomer 6 in their study (Sunahori et al., 2013). Kong et al. also identified isomer 6 in their work, but apparently did not consider it in their spectral assignment (Kong et al., 2006). Note that harmonic spectra were scaled by 0.9679 in the 1,000–2,000 cm^−1^ region and 0.95 in the 3,000–4,000 cm^−1^ region, as recommended by NIST and based on previous work for similar systems (Andersson and Uvdal, 2005; Fu and Hopkins, 2018).

It is necessary to highlight three caveats for the above example of identifying the spectral carrier of [Ser_2_ + H]^+^. First, to create the experimental spectrum that we used in our assignment, we collated the results of two separate studies (Sunahori et al., 2013; Seo et al., 2018). It is not necessarily true that the same ensemble populations were produced under the experimental conditions employed in both of these studies. However, given that isomer 6 provides the best match to both regions of the experimental spectrum, it seems to be that instrument conditions were similar in these two cases. A second consideration is the fact that peak intensities in IRMPD spectra are not necessarily well-modeled by computed absorption spectra owing to the fact that IRMPD intensities are dependent on absorption cross sections *and* the coupling efficiency for accessing dissociative channels. (Parneix et al., 2013) The methodology outline above assumes that the computed linear absorption intensities are representative of IRMPD intensities or, barring that, that the IRMPD intensities for a given band vary similarly from the computed intensity for all isomeric species. Finally, the above treatment also assumes that the computed harmonic frequencies suitably model the experimental spectrum. The validity of this assumption depends on the accuracy of the model chemistry and on the anharmonicity of the system being studied. While the [Ser_2_ + H]^+^ is apparently well-modeled by the B3LYP/6-311++G(d,p) + GD3 approach employed here, one should in general be aware of the anharmonic nature of hydrogen bonds and shared protons (Schofield et al., 2005; Oomens et al., 2009; Steill et al., 2011; Ieritano et al., 2016).

### Case Study 2: Dynamic Collision Cross Section of Protonated Alanine Tripeptide

Ion mobility spectrometry (IMS) is widely employed in the detection of illicit substances and for structural elucidation of ions (Collins and Lee, 2002; Verkouteren and Staymates, 2011; Lapthorn et al., 2013; Lanucara et al., 2014; Cumeras et al., 2015; Cajka and Fiehn, 2016; Paglia and Astarita, 2017). The success of IMS in determining analyte structure relies on accurate modeling of ion structure and subsequent calculation of CCSs for comparison with those determined experimentally. Experimental CCSs are obtained by relating the ion mobility, K, to CCS via the Mason-Schamp Equation (Mason and Mcdaniel, 1988; Ieritano et al., 2019b):

(12) $$\begin{array}{r} K=\frac{\sqrt{18\pi }}{16}\sqrt{\frac{1}{m_{ion}}+\frac{1}{m_{gas}}}\frac{ze}{\sqrt{k_{b}T}}\frac{1}{\Omega_{avg}}\frac{1}{N} \end{array}$$

Where *m*_*gas*_ is the mass of the buffer gas, *N* is the number density of the gas, *m*_*ion*_ is the mass of the ion, *z* is the ion charge state, *e* is the elementary charge, *k*_*b*_ is the Boltzmann constant, *T* is the temperature, and Ω_*avg*_ is the orientationally-averaged CCS. Typically, ion structures are viewed as rigid and ensembles are approximated as being composed of only a single structure in cases where multiple distinct signals are unresolved. This view is somewhat tenuous, particularly in the differential mobility spectrometry (DMS) variant of IMS wherein rapidly oscillating electric field conditions drive separations based on mobility differences between the high- and low-field portions of the applied waveform (Guevremont and Purves, 1999; Guevremont, 2004; Krylov et al., 2007, 2009; Krylov and Nazarov, 2009; Hopkins, 2015, 2019). The phenomenon of differential ion mobility is still not well-understood, and there is as yet no first principles model (Guevremont and Purves, 1999; Guevremont, 2004; Krylov et al., 2007, 2009; Krylov and Nazarov, 2009; Hopkins, 2015, 2019). However, one can view the effective temperature of an analyte ion in terms of the changing field conditions; the ion is relatively cold under low-field conditions and relatively hot under high-field conditions (Viehland and Mason, 1995; Robinson et al., 2008; Hopkins, 2019). By estimating ion temperatures with two-temperature theory (Robinson et al., 2008; Siems et al., 2016), we find that field-induced heating leads to effective ion temperature variations in the range of 300–800 K during one duty cycle of the commonly applied maximum electric field in the DMS cell (Hopkins, 2019). The variation in electric field, and therefore effective ion temperature, affects the ion mobility in two ways, the most obvious being the reduction of mobility with increasing temperature as predicted by Equation (12). Somewhat more subtle is the fact that Ω_*avg*_ must also be temperature-dependent since at elevated temperatures ions are able to access a larger region of the associated PES (assuming equipartition amongst the various DoFs of the molecule). Consequently, to accurately model an ion's Ω_*avg*_, one must identify which geometric structures are accessible under the given experimental conditions and estimate the contribution of that structure to the time-averaged CCS of the ion.

**Figure 8:**
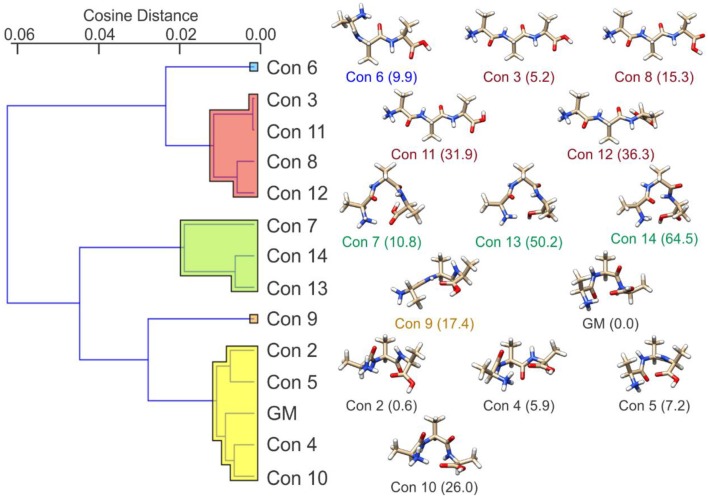
**(Left)** The cosine distance dendrogram for protonated alanine tripeptide, [AAA+H]^+^. **(Right)** Molecular geometries and relative standard Gibbs energies (kJ mol^−1^; in parentheses). Calculations were conducted at the B3LYP/6-311++G(d,p) + GD3 level of theory as implemented in Gaussian 16 (Frisch et al., 2016). Conformers are numbered in order of increasing energy relative to that of the global minimum (GM) structure.

If we consider the case of protonated alanine tripeptide, [AAA + H]^+^, there are several internal DoFs associated with dihedral angle rotations that can yield a variety of conformations. Upon application of the BH algorithm to search the PES of the [AAA + H]^+^ molecular ion, followed by re-optimization of the candidate structures at the B3LYP/6-31++G(d,p) + GD3 level of theory (Becke, 1988, 1993; Grimme et al., 2010), fourteen low energy conformations were identified. These structures are shown in Figure 8 along with their relative standard Gibbs energies (in kJ mol^−1^) (see sections 2.1 and 2.2 of the Supplementary Materials for details). Calculating the cosine distances between the various mass-weighted distance vectors and subsequent application of WPGMA agglomerative hierarchical clustering yields the dendrogram plot shown in Figure 8. Five unique sets of conformers are highlighted in the dendrogram. The set highlighted in yellow, of which the global minimum conformer is a member, contains compact structures that are stabilized by an IMHB between the protonated N-terminus and the carbonyl oxygen atom of the C-terminus. The set highlighted in green also contains relatively compact structures, but hydrogen bonding instead occurs between the protonated N-terminus and the hydroxyl group of the C-terminus. The set highlighted in red, on the other hand, contains elongated structures (i.e., the N- and C-termini do not interact). Conformers 6 and 9 (blue and orange, respectively) are intermediate species between the compact species (yellow and green sets) and the elongated species (red set). In the case of conformer 6, the N-terminus forms an IMHB with the nearest amide carbonyl rather than with the C-terminus. In contrast, the C-terminus of conformer 9 forms an IMHB with the most distant amino nitrogen instead of with the N-terminus.

If we calculate the relative Gibbs energies of the [AAA + H]^+^ conformers as a function of temperature, an interesting picture emerges. Owing to differences in the entropic contributions to the Gibbs energies, at low temperature the compact, H-bonded conformers associated with the yellow group are the dominant species in the ensemble, whereas at high temperature the elongated, non-H-bonded species in the red group dominate. One can estimate the relative populations of the various conformers via (Oh and Zeng, 1999; Vehkamäki, 2006; Hopkins, 2019):

(13) $$\begin{array}{r} N_{i}=N_{0}e^{-\frac{\Delta G_{rel}}{k_{B}T}} \end{array}$$

**Figure 9:**
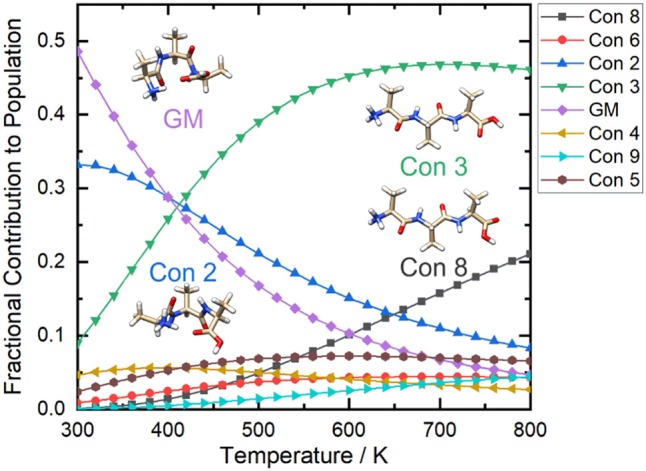
The relative populations of the low energy conformers of protonated alanine tripeptide, [AAA+H]^+^, as estimated via Gibbs energy calculations over the temperature range T = 300–800 K. Calculations were conducted at the B3LYP/6-311++G(d,p) + GD3 level of theory as implemented in Gaussian 16 (Frisch et al., 2016). Conformers are numbered in order of increasing energy relative to that of the global minimum (GM; i.e., conformer 1) structure.

Where *N*_0_ is the relative population of the lowest energy cluster (usually set to 1), *N*_*i*_ is the relative population of the *i*th cluster, Δ*G*_*rel*_ is the Gibbs energy of formation relative to the lowest energy cluster, and *k*_*B*_ is Boltzmann's constant. By calculating the relative populations of the clusters as a function of temperature (at a constant pressure of P = 1 atm), one can produce a temperature-dependent relative population plot as shown in Figure 9.

**Figure 10:**
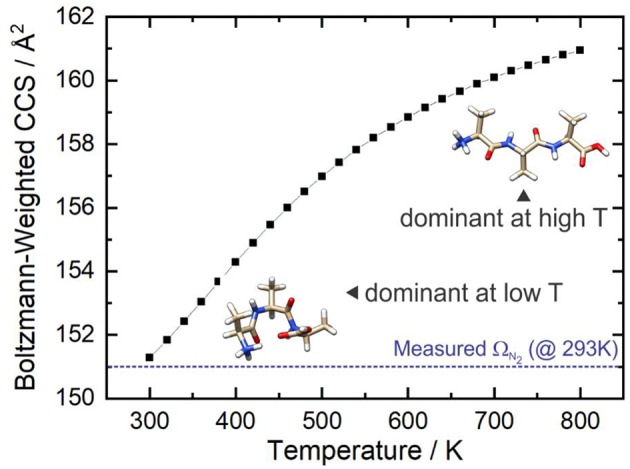
The Boltzmann-weighted CCS of [AAA + H]^+^ as a function of temperature at P = 1 atm. The dashed blue line shows the orientationally-averaged CCS, Ω_*ave*_, measured in N_2_ at room temperature (T ≈ 293 K) (Bush et al., 2012).

Figure 9 shows that at *ca*. T = 420 K [AAA + H]^+^ conformer 3 becomes the most populated species in the ensemble (i.e., the global minimum structure on the Gibbs energy surface). As the temperature increases further, conformer 3 is increasingly stabilized with respect to conformers 1 (the low T global minimum) and 2. At temperatures above 660 K, conformers 1 and 2 become minor contributors to the overall ensemble population in favor of conformers 3 and 8. This “tilting” of the Gibbs energy landscape as a function of temperature essentially decants the conformers associated with the yellow set into the red set (see Figure 8) as field-induced ion temperature increases, and back again as the temperature decreases during the low field portion of the oscillating DMS waveform. This dynamic process of peptide unfolding and re-folding yields a dynamic temperature-dependent ion CCS that, along with the effect of increased carrier gas viscosity at higher temperature (Mason and Mcdaniel, 1988; Hopkins, 2019), gives rise to differential mobility behavior. If one assumes that the ion quickly reaches thermal equilibrium, which is likely given the conditions of the DMS cell (1 atm of carrier gas), one can estimate the temperature-dependent ion CCS as a sum of the Boltzmann-weighted conformer CCSs (Ieritano et al., 2019a). This is plotted for [AAA + H]^+^ in Figure 10. It is worth noting that the experimentally-measured T ≈ 293 K value of Ω_*ave*_(N_2_) = 151 Å^2^ (Bush et al., 2012) is well-modeled by the T = 300 K Boltzmann-weighted sum of the various isomer CCSs as calculated using the MobCal-MPI code (https://uwaterloo.ca/hopkins-lab/mobcal-mpi), Ω_*Boltzmann*_(N_2_) = 151.3 Å^2^ (Ieritano et al., 2019b). In comparison, the calculated CCS for the static global minimum structure is Ω_*Boltzmann*_(N_2_) = 148.7 Å^2^. This demonstrates that even at a relatively low fixed temperature, there is some benefit in considering the relative populations of the conformeric species present in the experimental ensemble.

## Summary

Because the PESs of complex, fluxional molecular systems tend to be characterized by multiple funnels (*viz*. collections of closely related local minima), the BH framework has proven to be an effective search and optimization strategy (Locatelli, 2005; Olson et al., 2012). However, owing to the stochasticity of the algorithm, which is predominantly due to the random perturbative component, it is sometimes useful to introduce additional criteria which limit the regions of exploration on the PES. This has been traditionally accomplished by exploring specific degrees of freedom (e.g., dihedral rotations) on the potential energy landscape and by introducing a thermal energy distribution as a probabilistic means of accepting/rejecting random geometric perturbations. We have also introduced techniques from unsupervised machine learning, specifically distance matrices and hierarchical clustering, to further augment the BH algorithm. Although currently implemented as a separate module, these machine learning augmentations will in the future be incorporated for on-the-fly geometric analyses, which would ultimately provide additional control and efficiency during execution of the search algorithm afforded by reducing the search space to pertinent regions connecting known stationary points. This is particularly useful in identifying intermediate local minima and TSs between known isomers. Moreover, utilizing these same methods post-BH provides deep insights into the relation between stationary points and how these are partitioned on the potential energy landscape. This can be of great benefit in modeling experimental ensembles and in rationalizing the observation of kinetically-trapped species and dynamic molecular geometries.

In this manuscript we highlight the power of the BH framework in two case studies: (1) assigning the spectral carrier(s) of the IRMPD spectrum of [Ser_2_+H]^+^ and (2) modeling the temperature-dependent collision cross sections of [AAA+H]^+^. In case study 1, we show that a thorough mapping of the potential energy landscape is warranted to identify the species probed in gas phase ion spectroscopic studies of weakly-bound clusters. In the case of the protonated serine dimer, rather than observing the lowest energy isomer (as expected based on standard Gibbs energies), Seo et al. and Sunahori et al. observed a species that was associated with a relatively remote, higher energy region of the cluster PES (Sunahori et al., 2013; Seo et al., 2018). It is still an open question as to whether this was due to kinetic trapping during production or formation of this species *in situ* due to field-induced heating within the ion traps. In case study 2, we show that mapping PESs to identify low energy conformer geometries, which were subsequently refined at a higher level of quantum chemical theory, provides insight into how molecular geometry changes with increasing temperature. For [AAA+H]^+^, increasing the temperature of the system results in the dissociation of IMHBs and the formation of larger elongated structures compared to the compact H-bonded species favored at low temperature. We also demonstrate that modeling molecular collision cross sections as a Boltzmann-weighted sum of the CCSs for accessible conformers provides an accurate estimate of those measured experimentally (0.3 Å^2^ difference). It should be noted that this treatment assumes that the accessible conformers are readily interconvertible, and that thermal equilibrium is quickly established. In principle, one could also employ the interpolation techniques described in section Interpolating Intermediate Geometries to calculate barriers to interconversion and validate this assumption. However, the fact that our calculations yield results that are in excellent agreement with experimental measurements indicates that, in this case, the assumption is valid.

Ultimately, the BH framework is a useful approach to characterizing the structures and dynamics of chemical systems which exhibit PESs of high dimensionality. Examples of such systems range from weakly-bound nanoclusters to biological macromolecules. We note that, despite the success of our current implementation, the development of the BH framework by ourselves and others is ongoing. We expect that further tuning will improve general performance and, owing to the versatility of the method, that BH performance for specific tasks will continue to improve by tailoring key features of the algorithm.

## Author Contributions

CZ conducted the serine dimer work and wrote the original draft of manuscript. CI conducted the alanine tripeptide work. WH wrote the final draft of the manuscript.

### Conflict of Interest Statement

The authors declare that the research was conducted in the absence of any commercial or financial relationships that could be construed as a potential conflict of interest.

## Supplementary Material

Calculated energies and atomic XYZ coordinates for all species are available as Supplementary Material.
