# Supplementary material for: Augmenting Basin-Hopping With Techniques From Unsupervised Machine Learning: Applications in Spectroscopy and Ion Mobility
Source: Front Chem. 2019 Aug 7;7:519. doi: 10.3389/fchem.2019.00519 (PMC6693329; doi:10.3389/fchem.2019.00519)
Supplement: Supplementary file 1 [file Data_Sheet_1.docx]

Supplementary Material

**Augmenting Basin-Hopping with Techniques from Unsupervised Machine Learning: Applications in Spectroscopy and Ion Mobility**

Ce Zhou, Christian Ieritano, W. Scott Hopkins^*^

Department of Chemistry, University of Waterloo, 200 University Avenue West, Waterloo, Ontario, N2L 3G1, Canada

Table of Contents

[1. [Ser_2_H]^+^ Isomer 2](#_Toc7461591)

[1.1 Relative energies 2](#_Toc7461592)

[1.2 Isomer Geometries 3](#_Toc7461593)

[2. [AAA+H]^+^ Isomer 23](#_Toc7461594)

[2.1 Relative energies 23](#_Toc7461595)

[2.2 Isomer Geometries 24](#_Toc7461596)

# [Ser_2_H]^+^ Isomer

## Relative energies

| Isomer | Electronic Energy (Hartree) | Gibbs Energy at 298K (Hartree) | Rel Gibbs Energy at 298K (kJ mol^-1^) |
| --- | --- | --- | --- |
| 1 | -798.6307773 | -798.433571 | 0.00 |
| 2 | -798.629181 | -798.433136 | 1.14 |
| 3 | -798.6294905 | -798.432144 | 3.75 |
| 4 | -798.6283215 | -798.431752 | 4.78 |
| 5 | -798.6281536 | -798.431592 | 5.20 |
| 6 | -798.6260071 | -798.431451 | 5.57 |
| 7 | -798.6288936 | -798.430528 | 7.99 |
| 8 | -798.6254649 | -798.43024 | 8.75 |
| 9 | -798.627909 | -798.430057 | 9.23 |
| 10 | -798.6266171 | -798.428397 | 13.58 |
| 11 | -798.6230242 | -798.427932 | 14.81 |
| 12 | -798.6239287 | -798.427893 | 14.91 |
| 13 | -798.6240369 | -798.427849 | 15.02 |
| 14 | -798.6245727 | -798.427493 | 15.96 |
| 15 | -798.6235949 | -798.427484 | 15.98 |
| 16 | -798.6216751 | -798.426875 | 17.58 |
| 17 | -798.6226596 | -798.426836 | 17.68 |
| 18 | -798.6240105 | -798.426738 | 17.94 |
| 19 | -798.62362 | -798.426479 | 18.62 |
| 20 | -798.6186666 | -798.425654 | 20.79 |
| 21 | -798.617912 | -798.42546 | 21.30 |
| 22 | -798.6160862 | -798.424478 | 23.87 |
| 23 | -798.6187879 | -798.424285 | 24.38 |
| 24 | -798.620575 | -798.424226 | 24.54 |
| 25 | -798.6180663 | -798.424217 | 24.56 |
| 26 | -798.6175004 | -798.423944 | 25.28 |
| 27 | -798.6178296 | -798.423837 | 25.56 |
| 28 | -798.6163577 | -798.423624 | 26.12 |
| 29 | -798.618065 | -798.423523 | 26.38 |
| 30 | -798.6195043 | -798.422846 | 28.16 |
| 31 | -798.6205147 | -798.422833 | 28.19 |
| 32 | -798.6161979 | -798.422751 | 28.41 |
| 33 | -798.6164152 | -798.422553 | 28.93 |
| 34 | -798.6202486 | -798.421713 | 31.13 |
| 35 | -798.6165966 | -798.421613 | 31.40 |
| 36 | -798.6170171 | -798.421145 | 32.62 |
| 37 | -798.6162692 | -798.420925 | 33.20 |
| 38 | -798.6138529 | -798.418668 | 39.13 |
| 39 | -798.6156217 | -798.418609 | 39.28 |
| 40 | -798.6087986 | -798.413712 | 52.14 |

## Isomer Geometries

| Isomer 1 |  |  |  | Isomer 2 |  |  |  |
| --- | --- | --- | --- | --- | --- | --- | --- |
| Atom | x | y | z | Atom | x | y | z |
| C | -1.667870 | 0.109860 | 0.570224 | C | -1.729420 | 0.244308 | 0.166412 |
| H | -1.132804 | -0.378464 | 1.384872 | H | -1.161252 | 0.155359 | 1.092605 |
| C | -2.818902 | 0.956450 | 1.109307 | C | -2.874372 | 1.238195 | 0.361035 |
| H | -2.445626 | 1.636002 | 1.886108 | H | -3.479690 | 0.909391 | 1.209926 |
| H | -3.569036 | 0.294892 | 1.549607 | H | -3.513259 | 1.269490 | -0.532974 |
| O | -3.318358 | 1.675467 | -0.013752 | O | -2.242452 | 2.491505 | 0.590053 |
| H | -4.107757 | 2.173929 | 0.220884 | H | -2.897891 | 3.163431 | 0.805027 |
| C | -2.126111 | -0.983731 | -0.396481 | C | -2.184204 | -1.147931 | -0.244253 |
| O | -3.071223 | -1.741621 | 0.164320 | O | -3.062972 | -1.638435 | 0.631885 |
| H | -3.333271 | -2.438438 | -0.458724 | H | -3.326469 | -2.532410 | 0.358521 |
| O | -1.664060 | -1.144802 | -1.496033 | O | -1.785331 | -1.712877 | -1.230580 |
| H | 0.005929 | 0.428873 | -0.766805 | H | 0.029829 | 0.052417 | -1.067328 |
| H | -1.226169 | 1.619786 | -0.767442 | H | -1.260502 | 0.911895 | -1.755849 |
| H | -0.115004 | 1.530493 | 0.467509 | H | -0.349928 | 1.618670 | -0.562386 |
| N | -0.702313 | 0.980286 | -0.162892 | N | -0.780121 | 0.743354 | -0.870360 |
| C | 2.510830 | -0.285827 | -0.736835 | C | 2.481445 | -0.582422 | -0.488000 |
| H | 3.333673 | -0.876029 | -1.159178 | H | 3.336799 | -1.263246 | -0.585603 |
| C | 3.025565 | 1.147413 | -0.570417 | C | 2.988390 | 0.827808 | -0.801518 |
| H | 3.219304 | 1.577314 | -1.560106 | H | 3.306779 | 0.872631 | -1.849910 |
| H | 3.962039 | 1.122146 | -0.007404 | H | 3.849714 | 1.040626 | -0.163679 |
| O | 2.030562 | 1.915309 | 0.125308 | O | 1.924105 | 1.758672 | -0.557872 |
| H | 2.412186 | 2.762582 | 0.378213 | H | 2.289946 | 2.648108 | -0.510980 |
| C | 2.184251 | -0.876001 | 0.633018 | C | 2.004798 | -0.665442 | 0.959523 |
| O | 3.292761 | -1.057242 | 1.355767 | O | 3.007452 | -0.442697 | 1.813586 |
| H | 3.055148 | -1.423987 | 2.222421 | H | 2.673979 | -0.506231 | 2.722851 |
| O | 1.068231 | -1.136257 | 1.015899 | O | 0.867623 | -0.907211 | 1.287003 |
| N | 1.296907 | -0.311551 | -1.563236 | N | 1.359203 | -0.941704 | -1.367983 |
| H | 1.490322 | 0.050523 | -2.493137 | H | 1.663881 | -0.964109 | -2.337659 |
| H | 0.939024 | -1.256588 | -1.678890 | H | 0.997266 | -1.866335 | -1.145973 |

| Isomer 3 |  |  |  | Isomer 4 |  |  |  |
| --- | --- | --- | --- | --- | --- | --- | --- |
| Atom | x | y | z | Atom | x | y | z |
| C | -2.031321 | -0.446232 | -0.808869 | C | -2.421445 | -0.826341 | -0.416513 |
| H | -2.250493 | -0.425876 | -1.879553 | H | -3.316119 | -1.355898 | -0.764439 |
| C | -2.796888 | -1.594506 | -0.152501 | C | -2.154376 | -1.319680 | 1.010875 |
| H | -2.542729 | -2.537476 | -0.653526 | H | -1.787222 | -2.351464 | 0.975810 |
| H | -3.869043 | -1.413007 | -0.265068 | H | -3.096876 | -1.294860 | 1.563234 |
| O | -2.386078 | -1.598668 | 1.208948 | O | -1.187967 | -0.463889 | 1.643270 |
| H | -2.890506 | -2.248030 | 1.709672 | H | -1.156424 | -0.667542 | 2.583902 |
| C | -2.406763 | 0.919718 | -0.225447 | C | -2.752908 | 0.667656 | -0.380298 |
| O | -3.725848 | 1.111633 | -0.293309 | O | -3.959206 | 0.873753 | 0.158588 |
| H | -3.942782 | 1.985815 | 0.069075 | H | -4.134276 | 1.827943 | 0.193164 |
| O | -1.606769 | 1.710090 | 0.202957 | O | -2.017544 | 1.542096 | -0.767815 |
| H | 0.040565 | 0.218169 | -0.888049 | N | -1.262388 | -1.039111 | -1.300010 |
| H | -0.331344 | -0.757190 | 0.374386 | H | -0.917960 | -1.991618 | -1.211302 |
| H | -0.195436 | -1.430159 | -1.134483 | H | -1.526193 | -0.895603 | -2.271262 |
| N | -0.568392 | -0.630602 | -0.621509 | C | 2.091330 | 0.360121 | -0.404449 |
| C | 2.386541 | 0.952297 | -0.068340 | H | 2.326735 | 0.588704 | -1.447730 |
| H | 3.261460 | 1.607857 | -0.148756 | C | 2.931001 | 1.275929 | 0.490054 |
| C | 2.011066 | 0.907294 | 1.418086 | H | 3.986575 | 1.137977 | 0.241847 |
| H | 1.601061 | 1.879370 | 1.715198 | H | 2.780481 | 1.013840 | 1.546650 |
| H | 2.917449 | 0.711882 | 1.997250 | O | 2.467076 | 2.587656 | 0.206965 |
| O | 1.048285 | -0.134582 | 1.627319 | H | 2.994656 | 3.243204 | 0.675129 |
| H | 0.852399 | -0.208137 | 2.567398 | C | 2.345472 | -1.124408 | -0.170684 |
| C | 2.814972 | -0.445252 | -0.523118 | O | 3.648695 | -1.400293 | -0.262975 |
| O | 4.005079 | -0.768035 | -0.008331 | H | 3.789011 | -2.352204 | -0.130080 |
| H | 4.241888 | -1.666845 | -0.288225 | O | 1.474484 | -1.925362 | 0.053432 |
| O | 2.161495 | -1.168151 | -1.236695 | H | 0.313238 | 0.390815 | 0.740766 |
| N | 1.261802 | 1.406300 | -0.902903 | H | 0.432556 | 1.617353 | -0.374899 |
| H | 0.836434 | 2.241961 | -0.510020 | H | -0.003442 | 0.028395 | -0.810811 |
| H | 1.582239 | 1.633561 | -1.840397 | N | 0.644749 | 0.631911 | -0.205077 |

| Isomer 5 |  |  |  | Isomer 6 |  |  |  |
| --- | --- | --- | --- | --- | --- | --- | --- |
| Atom | x | y | z | Atom | x | y | z |
| C | -2.378386 | -0.233639 | 0.912042 | C | -1.989331 | -0.039771 | 0.181933 |
| H | -3.154568 | -0.687599 | 1.540123 | H | -1.242938 | -0.276574 | 0.943456 |
| C | -2.655807 | 1.273132 | 0.863898 | C | -2.250263 | 1.464679 | 0.174335 |
| H | -2.530450 | 1.691912 | 1.869890 | H | -1.293029 | 1.996035 | 0.136682 |
| H | -3.689164 | 1.432305 | 0.543799 | H | -2.777033 | 1.735051 | 1.092620 |
| O | -1.730231 | 1.867842 | -0.053354 | O | -3.032145 | 1.700116 | -0.992757 |
| H | -1.991185 | 2.779736 | -0.218635 | H | -3.283732 | 2.626973 | -1.059428 |
| C | -2.535089 | -0.821928 | -0.489352 | C | -3.232963 | -0.896969 | 0.435003 |
| O | -3.818561 | -0.838868 | -0.855410 | O | -3.901489 | -0.479148 | 1.507219 |
| H | -3.890542 | -1.196077 | -1.755313 | H | -4.665815 | -1.059662 | 1.657117 |
| O | -1.627884 | -1.212998 | -1.187585 | O | -3.514085 | -1.847998 | -0.247571 |
| N | -1.016766 | -0.488461 | 1.406124 | H | -1.684361 | -1.427320 | -1.335756 |
| H | -0.934990 | -0.182075 | 2.371594 | H | -1.900998 | 0.123897 | -1.874139 |
| H | -0.788756 | -1.480186 | 1.384222 | H | -0.392850 | -0.303812 | -1.195613 |
| C | 2.149316 | 0.132080 | -0.571716 | N | -1.442675 | -0.446726 | -1.153847 |
| H | 2.537186 | 0.121546 | -1.592714 | C | 3.323151 | 0.565802 | -0.205318 |
| C | 2.781271 | 1.311638 | 0.175415 | H | 3.573977 | 1.326989 | -0.950092 |
| H | 3.868715 | 1.219216 | 0.112968 | C | 4.025109 | -0.738440 | -0.585583 |
| H | 2.482338 | 1.294186 | 1.232064 | H | 5.103466 | -0.556480 | -0.656951 |
| O | 2.292565 | 2.473006 | -0.481945 | H | 3.651676 | -1.084185 | -1.554640 |
| H | 2.714345 | 3.263553 | -0.129353 | O | 3.717998 | -1.657326 | 0.460533 |
| C | 2.458245 | -1.201017 | 0.101053 | H | 4.180539 | -2.489242 | 0.318534 |
| O | 3.764914 | -1.465969 | 0.027577 | C | 1.799467 | 0.386520 | -0.286373 |
| H | 3.949173 | -2.313356 | 0.465343 | O | 1.141187 | 0.800105 | 0.781964 |
| O | 1.630516 | -1.894243 | 0.632628 | H | 1.867999 | 1.118339 | 1.396988 |
| H | 0.159637 | 0.101402 | 0.290727 | O | 1.226600 | -0.077789 | -1.259398 |
| H | 0.442312 | 1.281378 | -0.878546 | N | 3.642106 | 1.045764 | 1.144959 |
| H | 0.207913 | -0.316200 | -1.285959 | H | 4.025793 | 0.294903 | 1.712849 |
| N | 0.675365 | 0.316817 | -0.628057 | H | 4.285733 | 1.827209 | 1.146695 |

| Isomer 7 |  |  |  | Isomer 8 |  |  |  |
| --- | --- | --- | --- | --- | --- | --- | --- |
| Atom | x | y | z | Atom | x | y | z |
| C | 2.468093 | -0.824144 | 0.659395 | C | -3.166183 | -0.655775 | 0.246769 |
| H | 3.304144 | -1.045758 | 1.334423 | H | -3.326479 | -1.732458 | 0.315441 |
| C | 2.946142 | -1.129872 | -0.763619 | C | -4.020785 | -0.051884 | -0.862126 |
| H | 3.140209 | -2.205431 | -0.854829 | H | -5.036150 | -0.465242 | -0.843384 |
| H | 3.878700 | -0.588966 | -0.943701 | H | -3.545937 | -0.294343 | -1.815742 |
| O | 1.922387 | -0.718561 | -1.675877 | O | -4.036800 | 1.352261 | -0.602648 |
| H | 2.270664 | -0.733056 | -2.573675 | H | -4.512141 | 1.824555 | -1.293538 |
| C | 2.167431 | 0.667504 | 0.792600 | C | -1.635215 | -0.406506 | 0.081125 |
| O | 3.279930 | 1.390822 | 0.664905 | O | -1.043352 | 0.007628 | 1.101664 |
| H | 3.070219 | 2.334649 | 0.749057 | O | -1.164336 | -0.677656 | -1.045724 |
| O | 1.066412 | 1.133131 | 0.983858 | H | -3.943799 | 0.896278 | 1.386777 |
| N | 1.253520 | -1.593333 | 0.983176 | H | -4.125443 | -0.579784 | 2.169661 |
| H | 1.443946 | -2.588275 | 0.890229 | H | -2.567021 | 0.131139 | 1.999156 |
| H | 0.987480 | -1.437449 | 1.952646 | N | -3.517698 | -0.021198 | 1.572960 |
| C | -1.757327 | 0.227433 | 0.092728 | C | 1.970442 | 0.000030 | 0.135524 |
| H | -1.312282 | 0.430266 | 1.066526 | H | 1.180597 | 0.323207 | 0.820460 |
| C | -2.042602 | 1.570798 | -0.626358 | C | 2.437748 | -1.397511 | 0.527644 |
| H | -2.606348 | 2.217118 | 0.050915 | H | 1.565059 | -2.051712 | 0.637675 |
| H | -2.645263 | 1.398660 | -1.521250 | H | 2.965488 | -1.339136 | 1.482510 |
| O | -0.832959 | 2.151267 | -1.071815 | O | 3.284163 | -1.830662 | -0.534466 |
| H | -0.316695 | 2.451012 | -0.312045 | H | 3.646338 | -2.702890 | -0.349091 |
| C | -3.013325 | -0.613522 | 0.251754 | C | 3.075931 | 1.053762 | 0.124389 |
| O | -3.871115 | -0.033736 | 1.091726 | O | 3.229319 | 1.832947 | -0.781621 |
| H | -4.680245 | -0.568496 | 1.150488 | H | 1.914454 | -0.720414 | -1.799450 |
| O | -3.194901 | -1.647615 | -0.340203 | H | 0.334796 | -0.333466 | -1.197590 |
| H | -0.212147 | 0.079202 | -1.287007 | H | 1.483342 | 0.869809 | -1.682087 |
| H | -0.058031 | -1.044803 | -0.073208 | N | 1.380953 | -0.048440 | -1.238712 |
| H | -1.246696 | -1.250896 | -1.278141 | O | 3.792313 | 1.025662 | 1.249442 |
| N | -0.765828 | -0.555154 | -0.698915 | H | 4.462028 | 1.727788 | 1.216349 |

| Isomer 9 |  |  |  | Isomer 10 |  |  |  |
| --- | --- | --- | --- | --- | --- | --- | --- |
| Atom | x | y | z | Atom | x | y | z |
| C | -2.528918 | -0.653804 | -0.576986 | C | -2.715101 | 0.618842 | -0.128885 |
| H | -3.480429 | -0.998354 | -0.999838 | H | -3.784430 | 0.757484 | 0.083597 |
| C | -2.238015 | -1.534306 | 0.655266 | C | -2.631450 | -0.328262 | -1.346719 |
| H | -2.161293 | -2.578714 | 0.336133 | H | -3.070717 | 0.178075 | -2.209012 |
| H | -3.078003 | -1.453632 | 1.347088 | H | -3.210854 | -1.232992 | -1.151851 |
| O | -1.051345 | -1.119189 | 1.329302 | O | -1.281405 | -0.649618 | -1.696646 |
| H | -0.342609 | -1.752071 | 1.158758 | H | -1.011087 | -1.482122 | -1.287967 |
| C | -2.726825 | 0.793574 | -0.136904 | C | -2.110911 | -0.059677 | 1.094652 |
| O | -3.888089 | 0.941769 | 0.505525 | O | -2.920282 | -0.998342 | 1.583579 |
| H | -3.977714 | 1.862652 | 0.799335 | H | -2.494648 | -1.428360 | 2.343195 |
| O | -1.933979 | 1.686968 | -0.325171 | O | -1.018062 | 0.187882 | 1.564916 |
| N | -1.416673 | -0.716490 | -1.545950 | N | -1.990717 | 1.860445 | -0.406497 |
| H | -1.253457 | -1.683630 | -1.815829 | H | -2.385009 | 2.312476 | -1.226394 |
| H | -1.665750 | -0.215819 | -2.396205 | H | -2.089790 | 2.515284 | 0.364086 |
| C | 2.075294 | 0.356728 | -0.376143 | C | 2.087402 | 0.154445 | -0.454766 |
| H | 2.307065 | 0.551136 | -1.426530 | H | 2.541914 | 0.254221 | -1.444826 |
| C | 2.917881 | 1.305432 | 0.486142 | C | 2.975493 | 0.873979 | 0.563848 |
| H | 3.972615 | 1.160153 | 0.237669 | H | 3.984454 | 0.458453 | 0.508124 |
| H | 2.769163 | 1.080074 | 1.551148 | H | 2.584260 | 0.723078 | 1.578758 |
| O | 2.450096 | 2.603398 | 0.155425 | O | 2.924383 | 2.242509 | 0.182520 |
| H | 2.946216 | 3.277329 | 0.632309 | H | 3.507062 | 2.771621 | 0.737419 |
| C | 2.353339 | -1.110589 | -0.079875 | C | 1.900320 | -1.333009 | -0.175668 |
| O | 3.629495 | -1.401484 | -0.318424 | O | 3.073036 | -1.915114 | 0.071139 |
| H | 3.796654 | -2.338021 | -0.119621 | H | 2.941654 | -2.867627 | 0.211166 |
| O | 1.525553 | -1.894809 | 0.317466 | O | 0.835871 | -1.898855 | -0.200085 |
| H | 0.448147 | 1.644136 | -0.295510 | H | 0.240530 | 0.761849 | 0.399108 |
| H | -0.029571 | 0.110711 | -0.829915 | H | 0.868554 | 1.812692 | -0.720487 |
| H | 0.283477 | 0.370156 | 0.750559 | H | 0.084403 | 0.389773 | -1.175983 |
| N | 0.630259 | 0.643412 | -0.178557 | N | 0.755126 | 0.821053 | -0.503087 |

| Isomer 11 |  |  |  | Isomer 12 |  |  |  |
| --- | --- | --- | --- | --- | --- | --- | --- |
| Atom | x | y | z | Atom | x | y | z |
| N | 0.677878 | 0.081059 | -0.707808 | N | 1.299650 | 0.157014 | -1.195714 |
| H | 0.356273 | -0.661499 | -1.332824 | H | 1.685310 | 0.777403 | -1.912504 |
| C | 2.163883 | 0.157296 | -0.591275 | C | 2.403980 | -0.256030 | -0.284632 |
| H | 2.569961 | 0.204310 | -1.606111 | H | 3.102945 | -0.876242 | -0.852073 |
| C | 2.554981 | 1.438219 | 0.141804 | C | 3.147363 | 0.992118 | 0.202692 |
| C | 2.756258 | -1.079281 | 0.076653 | C | 1.822315 | -1.086813 | 0.851679 |
| H | 2.197955 | 1.407188 | 1.179706 | H | 2.502466 | 1.584145 | 0.865652 |
| H | 3.644971 | 1.508509 | 0.151768 | H | 4.032055 | 0.675073 | 0.760437 |
| O | 1.937975 | 2.490493 | -0.589686 | O | 3.471441 | 1.708221 | -0.981381 |
| O | 3.853115 | -1.100558 | 0.554725 | O | 0.651901 | -1.349044 | 0.956095 |
| H | 2.273476 | 3.343734 | -0.296698 | H | 4.025102 | 2.470291 | -0.780779 |
| O | 1.907522 | -2.126679 | 0.025767 | O | 2.785280 | -1.472132 | 1.689982 |
| H | 2.349405 | -2.907271 | 0.398645 | H | 2.402917 | -2.012891 | 2.400830 |
| H | 0.297764 | 0.977366 | -1.030427 | H | 0.475587 | 0.669736 | -0.696425 |
| H | 0.172695 | -0.116612 | 0.212091 | H | 0.825771 | -0.632191 | -1.646838 |
| N | -0.996855 | -0.544255 | 1.456366 | N | -1.007972 | 1.297675 | -0.208361 |
| H | -0.848496 | -0.150528 | 2.382023 | H | -1.376433 | 1.777044 | -1.030132 |
| C | -2.344148 | -0.186287 | 0.975911 | C | -1.954150 | 0.239739 | 0.199630 |
| H | -3.139974 | -0.540259 | 1.642649 | H | -1.557611 | -0.218180 | 1.110307 |
| C | -2.497173 | 1.333305 | 0.860648 | C | -3.353481 | 0.784380 | 0.518910 |
| C | -2.578837 | -0.834931 | -0.388212 | C | -1.975531 | -0.864487 | -0.845718 |
| H | -3.524487 | 1.560183 | 0.563123 | H | -3.816735 | 1.191447 | -0.391176 |
| H | -2.305102 | 1.789153 | 1.839726 | H | -3.982619 | -0.023985 | 0.901094 |
| O | -1.559354 | 1.804905 | -0.112546 | O | -3.141521 | 1.802413 | 1.490567 |
| O | -1.727146 | -1.385290 | -1.045591 | O | -1.223934 | -0.934802 | -1.794101 |
| H | -1.714137 | 2.740067 | -0.281852 | H | -3.985120 | 2.168014 | 1.775389 |
| O | -3.854507 | -0.709294 | -0.761082 | O | -2.921852 | -1.768964 | -0.586664 |
| H | -3.975951 | -1.116287 | -1.634126 | H | -2.887757 | -2.467196 | -1.260414 |
| H | -0.912139 | -1.554230 | 1.546533 | H | -1.001128 | 1.995727 | 0.533802 |

| Isomer 13 |  |  |  | Isomer 14 |  |  |  |
| --- | --- | --- | --- | --- | --- | --- | --- |
| Atom | x | y | z | Atom | x | y | z |
| C | 3.112914 | 0.562553 | -0.626457 | C | -3.052067 | -0.134573 | -0.035754 |
| H | 3.208218 | 1.215179 | -1.495279 | H | -3.934789 | -0.745668 | -0.246131 |
| C | 3.834449 | -0.759126 | -0.860460 | C | -2.642139 | 0.575853 | -1.327466 |
| H | 4.809527 | -0.597257 | -1.334753 | H | -3.398672 | 1.315265 | -1.598400 |
| H | 3.204662 | -1.365924 | -1.515469 | H | -2.536436 | -0.148697 | -2.137712 |
| O | 3.988940 | -1.338496 | 0.436831 | O | -1.369891 | 1.221125 | -1.078477 |
| H | 4.391895 | -2.210469 | 0.377325 | H | -1.311010 | 2.060107 | -1.548190 |
| C | 1.592199 | 0.401778 | -0.321751 | C | -1.927087 | -1.114497 | 0.361381 |
| O | 1.187708 | 1.102226 | 0.669703 | O | -1.568140 | -1.052892 | 1.633436 |
| H | 2.868986 | 1.653476 | 1.060747 | H | -2.170161 | -0.373787 | 2.036604 |
| O | 0.940106 | -0.327888 | -1.059255 | O | -1.373507 | -1.840149 | -0.439178 |
| N | 3.710146 | 1.265359 | 0.574899 | N | -3.319448 | 0.751347 | 1.099699 |
| H | 4.137318 | 0.549796 | 1.178688 | H | -2.854739 | 1.648213 | 0.997858 |
| H | 4.392533 | 1.988507 | 0.351215 | H | -4.306581 | 0.906823 | 1.262157 |
| C | -1.990640 | -0.008831 | -0.182761 | C | 1.587269 | -0.312718 | 0.260991 |
| H | -1.372988 | 0.182542 | -1.062820 | H | 0.860272 | -0.514722 | 1.051351 |
| C | -1.900186 | -1.491979 | 0.160283 | C | 2.758876 | -1.286412 | 0.360642 |
| H | -0.845877 | -1.784575 | 0.172077 | H | 2.379597 | -2.314536 | 0.405682 |
| H | -2.427233 | -2.064265 | -0.606633 | H | 3.316584 | -1.071946 | 1.275762 |
| O | -2.506954 | -1.623458 | 1.446495 | O | 3.528280 | -1.059673 | -0.814964 |
| H | -2.504520 | -2.542811 | 1.730598 | H | 4.324989 | -1.600425 | -0.816145 |
| C | -3.407727 | 0.498767 | -0.444632 | C | 2.005651 | 1.157695 | 0.333322 |
| O | -4.055990 | -0.279446 | -1.310722 | O | 2.724987 | 1.393278 | 1.430312 |
| H | -4.937453 | 0.092944 | -1.475332 | H | 2.975028 | 2.331305 | 1.457012 |
| O | -3.843222 | 1.507854 | 0.050598 | O | 1.686949 | 1.974861 | -0.490580 |
| H | -0.373495 | 0.918575 | 0.877265 | H | 1.603839 | -0.505901 | -1.803658 |
| H | -1.906859 | 1.705135 | 0.978809 | H | 0.360619 | -1.381010 | -1.052639 |
| H | -1.673543 | 0.306854 | 1.828944 | H | 0.206693 | 0.254505 | -1.218480 |
| N | -1.438992 | 0.792782 | 0.956543 | N | 0.901207 | -0.503349 | -1.058008 |

| Isomer 15 |  |  |  | Isomer 16 |  |  |  |
| --- | --- | --- | --- | --- | --- | --- | --- |
| Atom | x | y | z | Atom | x | y | z |
| C | 2.440986 | -0.006651 | 0.479451 | C | 1.533073 | 0.053462 | -0.185397 |
| H | 3.030040 | 0.228400 | 1.372883 | H | 0.796277 | -0.372854 | -0.868482 |
| C | 3.356745 | -0.758416 | -0.521048 | C | 1.762117 | 1.531550 | -0.491728 |
| H | 4.255476 | -0.166969 | -0.711152 | H | 0.797722 | 2.053850 | -0.529203 |
| H | 2.823337 | -0.890887 | -1.466950 | H | 2.252984 | 1.616560 | -1.464478 |
| O | 3.647923 | -2.057716 | -0.037921 | O | 2.572548 | 2.013496 | 0.573299 |
| H | 4.464578 | -2.051086 | 0.472231 | H | 2.861756 | 2.916585 | 0.407354 |
| C | 1.999493 | 1.302648 | -0.156022 | C | 2.803984 | -0.796904 | -0.266389 |
| O | 2.977931 | 2.210387 | -0.121017 | O | 3.422076 | -0.623545 | -1.435927 |
| H | 2.687874 | 3.018423 | -0.574560 | H | 4.210671 | -1.189636 | -1.468846 |
| O | 0.919213 | 1.491967 | -0.672730 | O | 3.150695 | -1.538922 | 0.613234 |
| N | 1.275122 | -0.847252 | 0.782384 | H | 1.033797 | -1.061421 | 1.490933 |
| H | 1.600067 | -1.808586 | 0.885466 | H | 1.576996 | 0.472647 | 1.834770 |
| H | 0.848850 | -0.590855 | 1.669563 | H | -0.028508 | 0.239064 | 1.247881 |
| C | -1.940243 | 0.357196 | -0.408023 | N | 0.993551 | -0.081613 | 1.200569 |
| H | -1.488797 | 1.346713 | -0.494057 | C | -2.607219 | 0.059976 | 0.353532 |
| C | -3.193319 | 0.256573 | -1.276085 | H | -3.647490 | 0.025215 | 0.691417 |
| H | -2.957497 | 0.564901 | -2.302785 | C | -2.565950 | 0.883376 | -0.945209 |
| H | -3.957261 | 0.926568 | -0.873777 | H | -3.309322 | 0.483856 | -1.640562 |
| O | -3.579828 | -1.111465 | -1.216733 | H | -1.571615 | 0.801696 | -1.408299 |
| H | -4.417285 | -1.253400 | -1.670090 | O | -2.837241 | 2.227285 | -0.575646 |
| C | -2.215847 | 0.088834 | 1.072253 | H | -3.131128 | 2.735095 | -1.338411 |
| O | -3.203700 | 0.866726 | 1.516874 | C | -2.146746 | -1.359680 | 0.042760 |
| H | -3.354317 | 0.687868 | 2.459398 | O | -3.130275 | -2.079008 | -0.500262 |
| O | -1.591816 | -0.696501 | 1.738192 | H | -2.800599 | -2.965668 | -0.720861 |
| H | -1.380619 | -1.558707 | -0.950040 | O | -1.021999 | -1.770597 | 0.221616 |
| H | -0.572646 | -0.370016 | -1.786635 | N | -1.719816 | 0.674065 | 1.359279 |
| H | -0.057906 | -0.717699 | -0.211398 | H | -1.887864 | 1.679591 | 1.343958 |
| N | -0.927775 | -0.643170 | -0.870180 | H | -1.991801 | 0.357913 | 2.287967 |

| Isomer 17 |  |  |  | Isomer 18 |  |  |  |
| --- | --- | --- | --- | --- | --- | --- | --- |
| Atom | x | y | z | Atom | x | y | z |
| C | -3.042877 | -0.319590 | -0.538280 | C | 2.405915 | 0.607603 | -0.577940 |
| H | -3.247576 | -0.584463 | -1.574485 | H | 3.369028 | 0.882783 | -1.029341 |
| C | -3.720574 | 1.004236 | -0.189042 | C | 2.362173 | 1.304670 | 0.798033 |
| H | -3.462175 | 1.731241 | -0.962006 | H | 2.443620 | 2.385323 | 0.651981 |
| H | -3.360947 | 1.374169 | 0.779920 | H | 3.211107 | 0.978659 | 1.400028 |
| O | -5.118214 | 0.720316 | -0.139270 | O | 1.163860 | 0.994360 | 1.521629 |
| H | -5.625504 | 1.521267 | 0.030385 | H | 0.533129 | 1.723198 | 1.455879 |
| C | -1.518521 | -0.256531 | -0.272676 | C | 2.438986 | -0.903088 | -0.371241 |
| O | -1.131231 | -0.962335 | 0.720703 | O | 3.657071 | -1.309594 | -0.006550 |
| H | -2.910570 | -1.540880 | 1.091868 | H | 3.648026 | -2.268476 | 0.144624 |
| O | -0.852982 | 0.474817 | -0.999189 | O | 1.489468 | -1.648698 | -0.488851 |
| N | -3.626421 | -1.400682 | 0.351290 | N | 1.237775 | 0.977169 | -1.376973 |
| H | -4.540951 | -1.101295 | 0.711401 | H | 1.230116 | 1.982533 | -1.525167 |
| H | -3.742991 | -2.284092 | -0.145142 | H | 1.291132 | 0.553074 | -2.298965 |
| C | 2.079912 | 0.032583 | -0.183828 | C | -1.514870 | -0.467497 | -0.310995 |
| H | 1.436225 | -0.135741 | -1.049791 | H | -0.822383 | -0.536712 | -1.149949 |
| C | 2.072760 | 1.522335 | 0.142815 | C | -2.721473 | -1.382611 | -0.494993 |
| H | 1.035652 | 1.870617 | 0.162571 | H | -2.380493 | -2.400291 | -0.723317 |
| H | 2.620431 | 2.057254 | -0.636437 | H | -3.319811 | -1.014654 | -1.332237 |
| O | 2.700073 | 1.635583 | 1.420875 | O | -3.422427 | -1.329820 | 0.742784 |
| H | 2.766174 | 2.558039 | 1.686728 | H | -4.241780 | -1.833572 | 0.697867 |
| C | 3.463201 | -0.551463 | -0.467062 | C | -1.887897 | 1.004089 | -0.145270 |
| O | 4.134660 | 0.182923 | -1.353111 | O | -2.657434 | 1.410920 | -1.153159 |
| H | 4.991827 | -0.237128 | -1.531025 | H | -2.883122 | 2.347102 | -1.028187 |
| O | 3.854044 | -1.577560 | 0.030687 | O | -1.510264 | 1.701044 | 0.763827 |
| H | 1.777131 | -0.238950 | 1.836763 | H | -1.405358 | -0.976457 | 1.700089 |
| H | 0.439885 | -0.813162 | 0.911772 | H | -0.291383 | -1.778960 | 0.732894 |
| H | 1.941492 | -1.657956 | 1.004932 | H | 0.002522 | -0.199401 | 1.143265 |
| N | 1.510377 | -0.727645 | 0.975199 | N | -0.758282 | -0.887004 | 0.910907 |

| Isomer 19 |  |  |  | Isomer 20 |  |  |  |
| --- | --- | --- | --- | --- | --- | --- | --- |
| Atom | x | y | z | Atom | x | y | z |
| N | 1.112690 | -0.734964 | 0.859172 | C | -3.357571 | -0.378917 | -0.410814 |
| H | 1.545033 | -1.568983 | 1.266494 | H | -3.494620 | -0.198082 | -1.484873 |
| C | 2.198110 | 0.206141 | 0.453291 | C | -4.096817 | 0.755133 | 0.318926 |
| H | 2.681627 | 0.577165 | 1.360427 | H | -3.994794 | 0.645610 | 1.405196 |
| C | 3.234517 | -0.559458 | -0.375734 | H | -5.152234 | 0.693277 | 0.051419 |
| C | 1.578683 | 1.379859 | -0.295264 | O | -3.524599 | 2.002783 | -0.120365 |
| H | 2.798485 | -0.869387 | -1.334584 | H | -3.985951 | 2.754235 | 0.267172 |
| H | 4.084679 | 0.100124 | -0.567104 | C | -1.828942 | -0.333067 | -0.204713 |
| O | 3.579124 | -1.680644 | 0.426797 | O | -1.223554 | 0.853172 | -0.218138 |
| O | 0.398882 | 1.490985 | -0.506489 | H | -1.906400 | 1.573159 | -0.248944 |
| H | 4.251163 | -2.218145 | -0.005897 | O | -1.163854 | -1.347795 | -0.072093 |
| O | 2.524509 | 2.242853 | -0.670011 | N | -3.972937 | -1.639275 | -0.072067 |
| H | 2.118320 | 2.988100 | -1.142657 | H | -3.713169 | -2.375493 | -0.716841 |
| H | 0.499313 | -1.024744 | 0.039235 | H | -3.727635 | -1.947489 | 0.862679 |
| H | 0.440871 | -0.321907 | 1.520543 | C | 2.000091 | 0.142407 | -0.013077 |
| N | -2.893668 | 0.067754 | -1.489825 | H | 1.235451 | 0.769912 | -0.476633 |
| H | -3.578255 | -0.379972 | -2.090509 | C | 2.164476 | 0.531479 | 1.454155 |
| C | -2.817731 | -0.608348 | -0.188228 | H | 1.178681 | 0.575781 | 1.931604 |
| H | -3.796539 | -0.934617 | 0.196280 | H | 2.633951 | 1.516577 | 1.507035 |
| C | -1.920699 | -1.838214 | -0.362843 | O | 2.975863 | -0.491570 | 2.021514 |
| C | -2.268567 | 0.333186 | 0.883158 | H | 3.176347 | -0.303965 | 2.944371 |
| H | -1.672641 | -2.290393 | 0.597282 | C | 3.283559 | 0.242869 | -0.842720 |
| H | -2.433747 | -2.582845 | -0.978603 | O | 3.878165 | 1.421481 | -0.677927 |
| O | -0.695428 | -1.448059 | -1.003638 | H | 4.670461 | 1.465250 | -1.238499 |
| O | -1.436008 | 0.033447 | 1.712861 | O | 3.651355 | -0.639651 | -1.574522 |
| H | -0.960778 | -0.763570 | -1.649287 | H | 1.967353 | -1.801570 | 0.686444 |
| O | -2.856411 | 1.529608 | 0.837071 | H | 0.489125 | -1.350023 | -0.048707 |
| H | -2.503299 | 2.090884 | 1.545715 | H | 1.893884 | -1.699366 | -0.964755 |
| H | -3.150605 | 1.044255 | -1.401801 | N | 1.543476 | -1.284239 | -0.092925 |

| Isomer 21 |  |  |  | Isomer 22 |  |  |  |
| --- | --- | --- | --- | --- | --- | --- | --- |
| Atom | x | y | z | Atom | x | y | z |
| C | -3.366556 | -0.372833 | -0.407689 | C | -3.217076 | -0.207471 | 0.717218 |
| H | -3.548454 | -0.186293 | -1.474261 | H | -3.702848 | -1.183402 | 0.848141 |
| C | -4.047589 | 0.770528 | 0.373751 | C | -3.996595 | 0.544267 | -0.382296 |
| H | -3.909313 | 0.633236 | 1.449407 | H | -3.554978 | 1.528651 | -0.557515 |
| H | -5.115348 | 0.743187 | 0.159059 | H | -5.022388 | 0.683675 | -0.042285 |
| O | -3.469812 | 2.046017 | 0.026145 | O | -3.945177 | -0.170179 | -1.633329 |
| H | -4.077833 | 2.565426 | -0.509338 | H | -4.779327 | -0.617696 | -1.807672 |
| C | -1.831472 | -0.344472 | -0.263227 | C | -1.778109 | -0.542505 | 0.281051 |
| O | -1.217421 | 0.836109 | -0.331002 | O | -1.576206 | -0.967075 | -0.961478 |
| H | -1.895821 | 1.561518 | -0.333945 | H | -2.413520 | -0.878354 | -1.486738 |
| O | -1.171344 | -1.361111 | -0.124280 | O | -0.827581 | -0.461315 | 1.046434 |
| N | -3.986867 | -1.624375 | -0.047916 | N | -3.320453 | 0.525894 | 1.955971 |
| H | -3.743337 | -2.370258 | -0.688305 | H | -3.004128 | -0.019102 | 2.749003 |
| H | -3.729823 | -1.924703 | 0.886407 | H | -2.779401 | 1.383945 | 1.936948 |
| C | 1.992672 | 0.140267 | -0.011792 | C | 2.549931 | -0.039407 | 0.297278 |
| H | 1.234387 | 0.770886 | -0.481493 | H | 2.471935 | -0.020190 | 1.385708 |
| C | 2.134003 | 0.515007 | 1.461624 | C | 3.932950 | -0.568635 | -0.101350 |
| H | 1.141292 | 0.555507 | 1.924981 | H | 4.692927 | 0.019124 | 0.420221 |
| H | 2.602727 | 1.499361 | 1.531493 | H | 4.083903 | -0.465886 | -1.184148 |
| O | 2.936264 | -0.513973 | 2.031427 | O | 3.926727 | -1.933821 | 0.294571 |
| H | 3.120299 | -0.336653 | 2.959748 | H | 4.780673 | -2.345621 | 0.123834 |
| C | 3.288667 | 0.250682 | -0.820522 | C | 2.274680 | 1.346958 | -0.272542 |
| O | 3.880127 | 1.427475 | -0.633527 | O | 3.071013 | 2.254430 | 0.291067 |
| H | 4.681187 | 1.477535 | -1.180934 | H | 2.901724 | 3.126703 | -0.102204 |
| O | 3.668014 | -0.623424 | -1.556668 | O | 1.459126 | 1.552206 | -1.133221 |
| H | 1.950111 | -1.809974 | 0.669142 | H | 1.281097 | -0.774263 | -1.194185 |
| H | 0.485536 | -1.355469 | -0.089835 | H | 1.820106 | -1.930243 | -0.103772 |
| H | 1.908088 | -1.692288 | -0.982338 | H | 0.574809 | -0.826533 | 0.318071 |
| N | 1.539913 | -1.286316 | -0.113428 | N | 1.490670 | -0.965038 | -0.209101 |

| Isomer 23 |  |  |  | Isomer 24 |  |  |  |
| --- | --- | --- | --- | --- | --- | --- | --- |
| Atom | x | y | z | Atom | x | y | z |
| C | 3.087537 | -0.332913 | 0.350634 | C | -2.861593 | 0.482314 | 0.023455 |
| H | 3.212249 | 0.291212 | 1.245009 | H | -3.285050 | 1.134979 | -0.747853 |
| C | 3.786766 | 0.409398 | -0.800679 | C | -1.883706 | 1.315658 | 0.879320 |
| H | 3.688657 | -0.152340 | -1.737143 | H | -1.249060 | 0.658453 | 1.475460 |
| H | 4.843575 | 0.500858 | -0.547486 | H | -2.471649 | 1.937812 | 1.560893 |
| O | 3.171149 | 1.707346 | -0.922178 | O | -1.001815 | 2.123849 | 0.093241 |
| H | 3.592848 | 2.227518 | -1.614552 | H | -1.430987 | 2.960122 | -0.114206 |
| C | 1.560897 | -0.432911 | 0.159243 | C | -2.161813 | -0.652078 | -0.756081 |
| O | 0.911958 | 0.625489 | -0.321336 | O | -2.852470 | -1.766168 | -0.840400 |
| H | 1.567003 | 1.313815 | -0.607682 | H | -3.672300 | -1.613758 | -0.308121 |
| O | 0.933474 | -1.433465 | 0.468852 | O | -1.067383 | -0.545096 | -1.284172 |
| N | 3.750156 | -1.595746 | 0.571481 | N | -3.967696 | -0.105906 | 0.785436 |
| H | 3.511744 | -1.997144 | 1.470001 | H | -3.704579 | -0.346941 | 1.736669 |
| H | 3.519068 | -2.279247 | -0.141616 | H | -4.785443 | 0.490303 | 0.821239 |
| C | -2.174195 | -0.074874 | -0.229195 | C | 2.184448 | -0.319469 | -0.682271 |
| H | -1.508430 | 0.054056 | -1.082267 | H | 2.102449 | -1.062366 | -1.478396 |
| C | -3.621989 | -0.215163 | -0.711447 | C | 3.605086 | 0.240578 | -0.623368 |
| H | -3.852085 | 0.628980 | -1.366955 | H | 3.899049 | 0.603050 | -1.617545 |
| H | -4.314369 | -0.202840 | 0.140570 | H | 4.285355 | -0.564158 | -0.330573 |
| O | -3.659215 | -1.463412 | -1.392075 | O | 3.557209 | 1.294891 | 0.326742 |
| H | -4.537313 | -1.633196 | -1.750055 | H | 4.440198 | 1.635433 | 0.503863 |
| C | -1.994184 | 1.085320 | 0.740497 | C | 1.761148 | -0.968249 | 0.637180 |
| O | -2.148923 | 2.252947 | 0.117621 | O | 2.547807 | -2.007829 | 0.915701 |
| H | -2.064691 | 2.975441 | 0.761661 | H | 2.271488 | -2.407301 | 1.756416 |
| O | -1.777668 | 0.929916 | 1.914052 | O | 0.841851 | -0.579905 | 1.308646 |
| H | -2.061996 | -1.300917 | 1.453887 | H | 1.165683 | 1.440541 | -0.180245 |
| H | -2.177051 | -2.129922 | -0.004493 | H | 1.460417 | 1.293871 | -1.803246 |
| H | -0.695631 | -1.415075 | 0.474553 | H | 0.228814 | 0.393198 | -1.075923 |
| N | -1.755693 | -1.327812 | 0.476057 | N | 1.208067 | 0.776807 | -0.960567 |

| Isomer 25 |  |  |  | Isomer 26 |  |  |  |
| --- | --- | --- | --- | --- | --- | --- | --- |
| Atom | x | y | z | Atom | x | y | z |
| C | -3.191286 | 0.601132 | 0.280626 | C | 3.119457 | -0.612192 | 0.002824 |
| H | -3.459457 | 1.305282 | 1.068609 | H | 3.673521 | -0.868320 | -0.903830 |
| C | -4.204258 | -0.548141 | 0.277729 | C | 1.917078 | -1.544824 | 0.138945 |
| H | -5.198492 | -0.148673 | 0.058728 | H | 1.354970 | -1.298322 | 1.044397 |
| H | -4.223470 | -1.057696 | 1.247842 | H | 2.281046 | -2.567909 | 0.237121 |
| O | -3.785328 | -1.454909 | -0.763089 | O | 1.014538 | -1.417490 | -0.977560 |
| H | -4.402965 | -2.187841 | -0.856941 | H | 1.099955 | -2.170763 | -1.570142 |
| C | -1.766796 | 0.136839 | 0.607949 | C | 2.695028 | 0.852959 | -0.115343 |
| O | -1.308518 | -0.951896 | -0.014180 | O | 3.682568 | 1.612975 | -0.585272 |
| H | -2.045117 | -1.396427 | -0.496437 | H | 3.415273 | 2.546343 | -0.569133 |
| O | -1.029415 | 0.761115 | 1.350327 | O | 1.622021 | 1.314427 | 0.239633 |
| N | -3.077506 | 1.312419 | -0.993176 | N | 3.999336 | -0.818927 | 1.147900 |
| H | -3.300418 | 0.715821 | -1.782099 | H | 4.923117 | -0.429018 | 1.004089 |
| H | -3.658928 | 2.139956 | -1.022906 | H | 3.616691 | -0.450221 | 2.012237 |
| C | 1.905150 | -0.125212 | -0.072208 | C | -1.955836 | 0.216655 | 0.533470 |
| H | 1.108447 | -0.802041 | -0.389689 | H | -1.467858 | -0.088066 | 1.462058 |
| C | 1.909905 | 1.123826 | -0.950624 | C | -2.757000 | 1.498480 | 0.758372 |
| H | 0.897615 | 1.542138 | -0.998158 | H | -2.114633 | 2.260652 | 1.216788 |
| H | 2.232741 | 0.845256 | -1.956740 | H | -3.588032 | 1.279585 | 1.433668 |
| O | 2.817925 | 2.018161 | -0.315307 | O | -3.193175 | 1.891221 | -0.536977 |
| H | 2.892756 | 2.841815 | -0.808197 | H | -3.777098 | 2.655197 | -0.488336 |
| C | 3.226220 | -0.897936 | -0.063588 | C | -2.811421 | -0.950399 | 0.027893 |
| O | 3.658623 | -1.133122 | -1.300686 | O | -3.840203 | -1.171624 | 0.847574 |
| H | 4.482307 | -1.646550 | -1.261669 | H | -4.361985 | -1.920342 | 0.515282 |
| O | 3.754727 | -1.267637 | 0.952746 | O | -2.546485 | -1.578657 | -0.961597 |
| H | 2.131591 | 1.156547 | 1.529841 | H | -1.328766 | 0.935254 | -1.299629 |
| H | 0.587998 | 0.439581 | 1.471246 | H | -0.118793 | 1.034233 | -0.130389 |
| H | 1.982293 | -0.429287 | 1.988427 | H | -0.445500 | -0.415009 | -0.816021 |
| N | 1.625416 | 0.282635 | 1.343225 | N | -0.902174 | 0.465473 | -0.495263 |

| Isomer 27 |  |  |  | Isomer 28 |  |  |  |
| --- | --- | --- | --- | --- | --- | --- | --- |
| Atom | x | y | z | Atom | x | y | z |
| C | 3.109101 | -0.326149 | 0.352044 | C | -2.845604 | 0.021301 | 0.812070 |
| H | 3.282840 | 0.299608 | 1.237341 | H | -2.836138 | -1.023574 | 1.148389 |
| C | 3.753592 | 0.397741 | -0.848759 | C | -4.140716 | 0.208091 | 0.004036 |
| H | 3.615111 | -0.182906 | -1.764390 | H | -4.208124 | 1.231567 | -0.383953 |
| H | 4.822536 | 0.490793 | -0.659816 | H | -4.982522 | 0.023187 | 0.672457 |
| O | 3.139942 | 1.685632 | -1.064019 | O | -4.120482 | -0.743699 | -1.076967 |
| H | 3.724643 | 2.396010 | -0.781693 | H | -4.922869 | -0.684790 | -1.606540 |
| C | 1.575141 | -0.407748 | 0.227105 | C | -1.577064 | 0.159829 | -0.051085 |
| O | 0.921778 | 0.662938 | -0.220002 | O | -1.575860 | -0.367426 | -1.269301 |
| H | 1.574039 | 1.341388 | -0.535935 | H | -2.482378 | -0.702545 | -1.491348 |
| O | 0.948198 | -1.403857 | 0.550820 | O | -0.563471 | 0.706080 | 0.363212 |
| N | 3.769239 | -1.593864 | 0.548948 | N | -2.881891 | 0.881188 | 1.971420 |
| H | 3.541502 | -2.007139 | 1.445141 | H | -2.193365 | 0.613220 | 2.663599 |
| H | 3.526141 | -2.266877 | -0.170335 | H | -2.716127 | 1.852808 | 1.731645 |
| C | -2.161165 | -0.089478 | -0.230465 | C | 2.745114 | -0.046750 | -0.510573 |
| H | -1.467366 | 0.028815 | -1.062496 | H | 3.557183 | -0.222682 | -1.221271 |
| C | -3.587078 | -0.275904 | -0.760709 | C | 3.112256 | 1.145819 | 0.373337 |
| H | -3.808711 | 0.540835 | -1.452749 | H | 4.068054 | 0.937210 | 0.860467 |
| H | -4.311022 | -0.249662 | 0.064323 | H | 2.343018 | 1.298772 | 1.140738 |
| O | -3.574248 | -1.546826 | -1.398875 | O | 3.181725 | 2.247053 | -0.524635 |
| H | -4.433466 | -1.745231 | -1.786689 | H | 3.437403 | 3.050904 | -0.059877 |
| C | -2.043010 | 1.103023 | 0.708952 | C | 2.471749 | -1.338084 | 0.252456 |
| O | -2.202802 | 2.247188 | 0.045287 | O | 3.477640 | -1.619885 | 1.081741 |
| H | -2.161528 | 2.990521 | 0.669385 | H | 3.299471 | -2.460631 | 1.534990 |
| O | -1.865967 | 0.988367 | 1.893834 | O | 1.479329 | -1.997238 | 0.089834 |
| H | -2.074839 | -1.255191 | 1.496394 | H | 0.685439 | 0.458871 | -0.630474 |
| H | -2.141430 | -2.135396 | 0.064482 | H | 1.679629 | 1.154731 | -1.818054 |
| H | -0.683181 | -1.385230 | 0.553375 | H | 1.233591 | -0.462215 | -1.915177 |
| N | -1.743520 | -1.311392 | 0.527953 | N | 1.512216 | 0.295219 | -1.288292 |

| Isomer 29 |  |  |  | Isomer 30 |  |  |  |
| --- | --- | --- | --- | --- | --- | --- | --- |
| Atom | x | y | z | Atom | x | y | z |
| C | 3.335260 | 0.336569 | -0.584310 | C | 2.941729 | -0.505800 | -0.341124 |
| H | 3.566741 | 0.673922 | -1.602490 | H | 2.580442 | -0.700449 | -1.356985 |
| C | 3.977339 | -1.039459 | -0.349980 | C | 2.193418 | -1.443497 | 0.629280 |
| H | 5.066509 | -0.953911 | -0.407540 | H | 2.379626 | -1.141559 | 1.662327 |
| H | 3.654285 | -1.758341 | -1.104121 | H | 2.578291 | -2.458939 | 0.499869 |
| O | 3.577780 | -1.581762 | 0.919650 | O | 0.769833 | -1.438824 | 0.464251 |
| H | 3.968828 | -1.038352 | 1.617779 | H | 0.498618 | -2.000463 | -0.272561 |
| C | 1.805565 | 0.281420 | -0.496079 | C | 2.624034 | 0.983598 | -0.074925 |
| O | 1.252244 | -0.604663 | 0.321922 | O | 3.651119 | 1.800421 | -0.218801 |
| H | 1.973253 | -1.137492 | 0.763797 | H | 4.428606 | 1.232075 | -0.427240 |
| O | 1.101843 | 1.047111 | -1.135611 | O | 1.515972 | 1.376985 | 0.222728 |
| N | 3.793398 | 1.248597 | 0.474241 | N | 4.394090 | -0.691228 | -0.330606 |
| H | 4.789709 | 1.424381 | 0.375313 | H | 4.756673 | -0.939293 | 0.585267 |
| H | 3.331475 | 2.150792 | 0.413993 | H | 4.714417 | -1.379348 | -1.000782 |
| C | -1.971888 | -0.144139 | 0.036495 | C | -2.387569 | 0.188703 | 0.725325 |
| H | -1.205842 | -0.922037 | 0.011779 | H | -2.468636 | -0.156933 | 1.758454 |
| C | -2.004730 | 0.497536 | 1.421592 | C | -3.369207 | 1.333395 | 0.472009 |
| H | -0.984945 | 0.762482 | 1.723067 | H | -3.212048 | 2.126051 | 1.214832 |
| H | -2.414061 | -0.222819 | 2.133770 | H | -4.388864 | 0.952406 | 0.572382 |
| O | -2.827374 | 1.651049 | 1.276994 | O | -3.077856 | 1.780419 | -0.844736 |
| H | -2.954386 | 2.095003 | 2.121826 | H | -3.680318 | 2.482138 | -1.112805 |
| C | -3.302869 | -0.753683 | -0.413460 | C | -2.625297 | -1.008184 | -0.201815 |
| O | -3.812445 | -1.551560 | 0.521067 | O | -3.882124 | -1.435696 | -0.102165 |
| H | -4.637871 | -1.946507 | 0.194627 | H | -4.014649 | -2.196578 | -0.691051 |
| O | -3.773787 | -0.546691 | -1.502067 | O | -1.771240 | -1.493520 | -0.898606 |
| H | -2.028679 | 1.791265 | -0.680588 | H | -0.927258 | 1.115292 | -0.429796 |
| H | -0.562407 | 1.003399 | -1.060627 | H | -0.685534 | 1.360186 | 1.168261 |
| H | -2.025057 | 0.651225 | -1.882347 | H | -0.274855 | -0.106746 | 0.495764 |
| N | -1.609928 | 0.901353 | -0.977017 | N | -0.993058 | 0.663312 | 0.487778 |

| Isomer 31 |  |  |  | Isomer 32 |  |  |  |
| --- | --- | --- | --- | --- | --- | --- | --- |
| Atom | x | y | z | Atom | x | y | z |
| C | -2.323228 | -0.514773 | -0.671863 | N | 0.968141 | 0.158965 | 0.611904 |
| H | -3.275517 | -0.674902 | -1.194992 | H | 1.261444 | 0.112590 | 1.592066 |
| C | -2.433191 | -1.255111 | 0.677181 | C | 2.159732 | 0.179333 | -0.288260 |
| H | -2.610280 | -2.318048 | 0.490823 | H | 1.839308 | -0.181874 | -1.266884 |
| H | -3.278179 | -0.864441 | 1.244869 | C | 2.679399 | 1.613258 | -0.431593 |
| O | -1.254333 | -1.085063 | 1.476411 | C | 3.208046 | -0.759481 | 0.298777 |
| H | -0.663677 | -1.840602 | 1.357873 | H | 3.088449 | 1.966534 | 0.524632 |
| C | -2.214806 | 0.980963 | -0.397540 | H | 3.476693 | 1.618050 | -1.180068 |
| O | -3.413111 | 1.507161 | -0.129787 | O | 1.548080 | 2.373139 | -0.828987 |
| H | -3.313519 | 2.450545 | 0.076510 | O | 3.110074 | -1.242847 | 1.396760 |
| O | -1.182280 | 1.615394 | -0.380145 | H | 1.783356 | 3.300573 | -0.940324 |
| N | -1.144019 | -0.976706 | -1.405810 | O | 4.220111 | -0.930297 | -0.549781 |
| H | -1.246412 | -1.963629 | -1.626154 | H | 4.888287 | -1.511581 | -0.149507 |
| H | -1.067046 | -0.492783 | -2.296025 | H | 0.350869 | -0.663244 | 0.417850 |
| C | 1.618261 | 0.273591 | -0.051473 | H | 0.370876 | 0.984584 | 0.462462 |
| H | 0.946625 | 0.546883 | -0.864892 | N | -4.018459 | -0.139621 | -1.326878 |
| C | 2.920175 | 1.092561 | -0.114074 | H | -4.942781 | -0.019123 | -0.930168 |
| H | 3.379973 | 0.956200 | -1.094239 | C | -3.060146 | -0.655708 | -0.355828 |
| H | 3.627451 | 0.740387 | 0.643876 | H | -3.529207 | -1.495181 | 0.165980 |
| O | 2.614721 | 2.447071 | 0.182192 | C | -1.828197 | -1.150707 | -1.112928 |
| H | 2.599308 | 2.979057 | -0.619772 | C | -2.690628 | 0.394091 | 0.695507 |
| C | 1.823695 | -1.229141 | -0.091080 | H | -2.140599 | -1.879091 | -1.862416 |
| O | 2.498894 | -1.582662 | -1.183513 | H | -1.344463 | -0.316490 | -1.625158 |
| H | 2.633397 | -2.544780 | -1.185238 | O | -0.825948 | -1.714701 | -0.239990 |
| O | 1.436831 | -1.986530 | 0.766154 | O | -1.659980 | 1.038431 | 0.717648 |
| H | -0.030217 | 0.109731 | 1.259971 | H | -1.049503 | -2.621775 | -0.005059 |
| H | 1.431850 | 0.372872 | 2.036304 | O | -3.683696 | 0.576620 | 1.571009 |
| H | 0.718442 | 1.630294 | 1.204755 | H | -3.456688 | 1.303771 | 2.172698 |
| N | 0.886392 | 0.619807 | 1.207085 | H | -3.722182 | 0.739365 | -1.738612 |

| Isomer 33 |  |  |  | Isomer 34 |  |  |  |
| --- | --- | --- | --- | --- | --- | --- | --- |
| Atom | x | y | z | Atom | x | y | z |
| C | -3.210038 | 0.335046 | -0.151493 | C | -2.838282 | -0.235780 | 0.177348 |
| H | -3.617703 | 0.541199 | -1.145172 | H | -3.389558 | -1.007876 | 0.724023 |
| C | -2.402123 | 1.541580 | 0.324182 | C | -2.271098 | -0.844998 | -1.130304 |
| H | -1.975959 | 1.341363 | 1.310277 | H | -1.757169 | -0.072597 | -1.702815 |
| H | -3.070111 | 2.398865 | 0.414605 | H | -3.102168 | -1.232694 | -1.728187 |
| O | -1.293436 | 1.839755 | -0.552368 | O | -1.299693 | -1.866717 | -0.906063 |
| H | -1.564061 | 2.470147 | -1.227476 | H | -1.728636 | -2.648183 | -0.538042 |
| C | -2.344540 | -0.922373 | -0.261413 | C | -1.662501 | 0.195273 | 1.063567 |
| O | -2.952037 | -1.871275 | -0.975785 | O | -1.382937 | 1.494757 | 1.017906 |
| H | -2.426378 | -2.687189 | -0.948206 | H | -2.112699 | 1.903965 | 0.491705 |
| O | -1.266092 | -1.094990 | 0.277822 | O | -0.985185 | -0.587995 | 1.693131 |
| N | -4.328959 | 0.140349 | 0.763411 | N | -3.644646 | 0.941806 | -0.155666 |
| H | -5.037170 | -0.476802 | 0.384298 | H | -3.998487 | 0.929702 | -1.106354 |
| H | -4.039616 | -0.210208 | 1.670724 | H | -4.435454 | 1.058669 | 0.468562 |
| C | 1.956357 | 0.077282 | -0.464070 | C | 1.733148 | 0.032271 | 0.520010 |
| H | 1.451732 | -0.289386 | -1.360202 | H | 1.169971 | 0.439406 | 1.362066 |
| C | 2.914218 | 1.213312 | -0.820387 | C | 3.223997 | -0.039410 | 0.838348 |
| H | 2.382647 | 1.978593 | -1.400319 | H | 3.373908 | -0.528390 | 1.808893 |
| H | 3.725421 | 0.806835 | -1.430148 | H | 3.623348 | 0.975999 | 0.888452 |
| O | 3.366823 | 1.722567 | 0.426753 | O | 3.794255 | -0.800803 | -0.222149 |
| H | 4.067661 | 2.370845 | 0.302380 | H | 4.752993 | -0.842582 | -0.142575 |
| C | 2.656656 | -1.095891 | 0.228479 | C | 1.389062 | 0.879748 | -0.704851 |
| O | 3.623505 | -1.590572 | -0.547483 | O | 2.020637 | 2.050201 | -0.668161 |
| H | 4.054098 | -2.332208 | -0.091522 | H | 1.754233 | 2.585113 | -1.433423 |
| O | 2.341518 | -1.498833 | 1.314713 | O | 0.606738 | 0.528679 | -1.550572 |
| H | 1.377676 | 0.984042 | 1.304298 | H | 1.943914 | -1.876707 | -0.255363 |
| H | 0.293283 | 1.282919 | 0.035129 | H | 1.034769 | -1.827197 | 1.155807 |
| H | 0.282281 | -0.181945 | 0.770815 | H | 0.327459 | -1.386645 | -0.269097 |
| N | 0.916212 | 0.581934 | 0.483661 | N | 1.225662 | -1.361463 | 0.266939 |

| Isomer 35 |  |  |  | Isomer 36 |  |  |  |
| --- | --- | --- | --- | --- | --- | --- | --- |
| Atom | x | y | z | Atom | x | y | z |
| C | -2.838576 | -0.667641 | -0.214147 | C | -2.834244 | -0.220319 | -0.638961 |
| H | -3.130667 | -1.007182 | -1.216329 | H | -3.521731 | -1.069726 | -0.687327 |
| C | -3.423372 | 0.748516 | -0.023907 | C | -1.556918 | -0.547749 | -1.411376 |
| H | -3.156189 | 1.141409 | 0.960182 | H | -0.870780 | 0.299065 | -1.379650 |
| H | -4.509117 | 0.683173 | -0.086101 | H | -1.822623 | -0.731788 | -2.453370 |
| O | -2.891817 | 1.665203 | -1.004204 | O | -0.850486 | -1.672226 | -0.847920 |
| H | -3.543934 | 1.848918 | -1.688056 | H | -1.120183 | -2.483911 | -1.288277 |
| C | -1.297365 | -0.652691 | -0.228768 | C | -2.567969 | 0.057332 | 0.842030 |
| O | -0.689328 | 0.313138 | -0.912956 | O | -3.698580 | 0.051993 | 1.548027 |
| H | -1.362678 | 0.978125 | -1.211964 | H | -3.512079 | 0.310247 | 2.465130 |
| O | -0.633449 | -1.502690 | 0.341743 | O | -1.490634 | 0.326243 | 1.351072 |
| N | -3.426619 | -1.553079 | 0.761559 | N | -3.478716 | 0.919336 | -1.282821 |
| H | -3.250498 | -2.527357 | 0.548004 | H | -4.424442 | 1.070566 | -0.952944 |
| H | -3.075827 | -1.374822 | 1.696768 | H | -2.948814 | 1.778485 | -1.178657 |
| C | 2.562123 | 0.000463 | 0.368065 | C | 2.273775 | 0.078179 | 0.880393 |
| H | 3.573135 | 0.107268 | 0.771838 | H | 2.423242 | 0.527924 | 1.864963 |
| C | 2.658548 | -0.170762 | -1.147585 | C | 3.435078 | -0.860766 | 0.555788 |
| H | 3.206122 | 0.681938 | -1.557042 | H | 3.557345 | -1.591703 | 1.366426 |
| H | 1.657966 | -0.202773 | -1.589639 | H | 4.350054 | -0.266986 | 0.480044 |
| O | 3.355483 | -1.402040 | -1.318321 | O | 3.083263 | -1.488201 | -0.667874 |
| H | 3.487689 | -1.592899 | -2.252854 | H | 3.816399 | -2.013423 | -1.005095 |
| C | 1.731068 | 1.185684 | 0.845167 | C | 2.120472 | 1.204598 | -0.144180 |
| O | 2.055231 | 2.294428 | 0.180183 | O | 3.249346 | 1.907787 | -0.236290 |
| H | 1.549504 | 3.043767 | 0.535561 | H | 3.132929 | 2.623710 | -0.882146 |
| O | 0.925303 | 1.100694 | 1.734087 | O | 1.104586 | 1.418404 | -0.750074 |
| H | 0.953013 | -1.358427 | 0.660459 | H | 1.001724 | -1.402703 | 1.627338 |
| H | 2.506277 | -2.050609 | 0.630941 | H | 0.135403 | -0.104095 | 1.043540 |
| H | 1.974562 | -1.205588 | 1.981978 | H | 0.806906 | -1.175589 | 0.001184 |
| N | 1.968718 | -1.243523 | 0.961163 | N | 0.992362 | -0.689296 | 0.897272 |

| Isomer 37 |  |  |  | Isomer 38 |  |  |  |
| --- | --- | --- | --- | --- | --- | --- | --- |
| Atom | x | y | z | Atom | x | y | z |
| C | -2.978879 | -0.422471 | 0.079276 | N | -3.164768 | 1.237977 | -0.951333 |
| H | -3.721737 | -0.403743 | 0.881901 | H | -3.032624 | 2.221932 | -0.715674 |
| C | -1.848896 | -1.384254 | 0.446241 | C | -2.901373 | 0.328151 | 0.229009 |
| H | -1.095123 | -1.390635 | -0.343564 | H | -3.041462 | 0.901060 | 1.144270 |
| H | -2.259894 | -2.390243 | 0.544024 | C | -3.894058 | -0.832923 | 0.171813 |
| O | -1.152046 | -0.999104 | 1.652335 | C | -1.443174 | -0.165588 | 0.084830 |
| H | -1.670460 | -1.229173 | 2.430975 | H | -3.626779 | -1.522726 | -0.638937 |
| C | -2.464966 | 1.005571 | -0.115215 | H | -3.846489 | -1.368856 | 1.122560 |
| O | -3.459417 | 1.895265 | -0.061956 | O | -5.167775 | -0.236134 | -0.063989 |
| H | -3.116637 | 2.778242 | -0.275909 | O | -0.884297 | 0.070666 | -1.000777 |
| O | -1.313939 | 1.314498 | -0.357390 | H | -5.862199 | -0.903532 | -0.080422 |
| N | -3.632355 | -0.925518 | -1.125191 | O | -0.987775 | -0.784828 | 1.083212 |
| H | -4.533620 | -0.491281 | -1.285238 | H | 0.403711 | -1.185537 | 0.862991 |
| H | -3.061065 | -0.807436 | -1.955653 | H | -4.125126 | 1.103154 | -1.290144 |
| C | 2.245473 | -0.307403 | 0.646474 | H | -2.435665 | 0.975242 | -1.647234 |
| H | 2.638748 | -0.603004 | 1.623134 | N | 1.487405 | -1.366780 | 0.660374 |
| C | 1.923620 | -1.555543 | -0.172714 | H | 1.583987 | -2.147259 | 0.008994 |
| H | 1.294643 | -2.230080 | 0.419985 | C | 2.073030 | -0.115184 | 0.077129 |
| H | 2.861007 | -2.062837 | -0.409428 | H | 1.421382 | 0.159156 | -0.758055 |
| O | 1.232047 | -1.106927 | -1.332962 | C | 2.023452 | 1.031705 | 1.108383 |
| H | 1.373377 | -1.710326 | -2.069022 | C | 3.477694 | -0.327839 | -0.466667 |
| C | 3.262954 | 0.633121 | -0.007538 | H | 1.039015 | 1.022615 | 1.581081 |
| O | 4.334180 | -0.035283 | -0.434406 | H | 2.779582 | 0.866935 | 1.888991 |
| H | 4.976004 | 0.590129 | -0.809606 | O | 2.167124 | 2.282493 | 0.485420 |
| O | 3.096696 | 1.821907 | -0.073020 | O | 4.277146 | 0.559650 | -0.590985 |
| H | 0.512917 | 0.697276 | -0.025186 | H | 3.074315 | 2.366398 | 0.163888 |
| H | 0.285666 | -0.025400 | 1.422324 | O | 3.701387 | -1.612839 | -0.809376 |
| H | 1.212754 | 1.395603 | 1.285783 | H | 4.592723 | -1.693591 | -1.187059 |
| N | 0.999128 | 0.489224 | 0.863123 | H | 1.957264 | -1.635374 | 1.526445 |

| Isomer 39 |  |  |  | Isomer 40 |  |  |  |
| --- | --- | --- | --- | --- | --- | --- | --- |
| Atom | x | y | z | Atom | x | y | z |
| N | -2.121733 | 1.361740 | -0.290900 | N | -0.975322 | -0.358491 | 1.127340 |
| H | -1.222729 | 1.823565 | -0.071176 | H | -1.243139 | -1.141121 | 1.726600 |
| C | -2.074845 | -0.047751 | 0.241319 | C | -2.173726 | 0.403392 | 0.655644 |
| H | -1.783077 | 0.020303 | 1.287879 | H | -2.685942 | 0.805450 | 1.532798 |
| C | -3.462333 | -0.671211 | 0.110955 | C | -1.716400 | 1.561117 | -0.236180 |
| C | -1.000654 | -0.762668 | -0.581232 | C | -3.089750 | -0.582882 | -0.063029 |
| H | -3.703704 | -0.844916 | -0.945415 | H | -1.315096 | 1.173834 | -1.180875 |
| H | -3.463100 | -1.630328 | 0.637748 | H | -2.582135 | 2.191138 | -0.451627 |
| O | -4.344230 | 0.277864 | 0.699693 | O | -0.709606 | 2.239521 | 0.505980 |
| O | -0.814459 | -0.467854 | -1.737075 | O | -2.837919 | -1.755246 | -0.160067 |
| H | -5.246623 | -0.058383 | 0.725311 | H | -0.598538 | 3.140378 | 0.184092 |
| O | -0.345433 | -1.658247 | 0.130476 | O | -4.165679 | 0.037149 | -0.544385 |
| H | 0.517461 | -1.877590 | -0.315796 | H | -4.745736 | -0.605419 | -0.986478 |
| H | -2.924082 | 1.854836 | 0.113754 | H | -0.319076 | 0.248573 | 1.630397 |
| H | -2.207158 | 1.330821 | -1.313417 | H | -0.440125 | -0.759806 | 0.312247 |
| N | 1.911487 | -0.388516 | 1.987147 | N | 3.240877 | 1.179551 | -1.194262 |
| H | 1.049000 | -0.852474 | 1.731215 | H | 4.068398 | 0.605217 | -1.328817 |
| C | 2.583806 | 0.209650 | 0.859136 | C | 2.180849 | 0.424689 | -0.547784 |
| H | 3.443409 | 0.778689 | 1.232996 | H | 1.397898 | 1.117779 | -0.229891 |
| C | 3.187388 | -0.847592 | -0.074288 | C | 1.599161 | -0.542524 | -1.585158 |
| C | 1.750491 | 1.226558 | 0.060045 | C | 2.673299 | -0.369541 | 0.668790 |
| H | 3.829829 | -0.365211 | -0.813639 | H | 1.293634 | 0.031697 | -2.461879 |
| H | 3.768960 | -1.540485 | 0.537341 | H | 2.358441 | -1.274281 | -1.874616 |
| O | 2.127647 | -1.556777 | -0.756393 | O | 0.459156 | -1.222440 | -1.005958 |
| O | 0.581612 | 1.492190 | 0.278050 | O | 3.689494 | -1.002128 | 0.698828 |
| H | 2.497820 | -2.073734 | -1.479858 | H | 0.229084 | -2.004518 | -1.519790 |
| O | 2.466654 | 1.817366 | -0.894912 | O | 1.830150 | -0.279787 | 1.747089 |
| H | 1.922023 | 2.461184 | -1.374269 | H | 2.244948 | -0.767179 | 2.478690 |
| H | 1.716241 | 0.282903 | 2.719261 | H | 3.513146 | 1.988782 | -0.647992 |

# [AAA+H]^+^ Isomer

## Relative energies

| Isomer | Electronic Energy (Hartree) | Gibbs Energy at 298K (Hartree) | Rel Gibbs Energy at 298K (kJ mol^-1^) |
| --- | --- | --- | --- |
| 1 | -819.0788946 | -818.832454 | 0.00 |
| 2 | -819.0711902 | -818.830473 | 5.20 |
| 3 | -819.0760775 | -818.830199 | 5.92 |
| 4 | -819.0735511 | -818.829697 | 7.24 |
| 5 | -819.0725644 | -818.828677 | 9.92 |
| 6 | -819.0755759 | -818.828335 | 10.81 |
| 7 | -819.0660066 | -818.826633 | 15.28 |
| 8 | -819.0678388 | -818.825829 | 17.39 |
| 9 | -819.0669579 | -818.822568 | 25.96 |
| 10 | -819.0605888 | -818.820316 | 31.87 |
| 11 | -819.0601429 | -818.818642 | 36.26 |
| 12 | -819.0582356 | -818.813342 | 50.18 |
| 13 | -819.0582354 | -818.813338 | 50.19 |
| 14 | -819.0513912 | -818.807893 | 64.48 |

## Isomer Geometries

| Isomer 1 |  |  |  | Isomer 2 |  |  |  |
| --- | --- | --- | --- | --- | --- | --- | --- |
| Atom | x | y | z | Atom | x | y | z |
| N | -2.009783 | -2.199675 | -0.651567 | N | -5.306693 | -0.206421 | 0.159675 |
| H | -1.016099 | -2.232818 | -0.332357 | H | -5.867568 | 0.367260 | 0.790759 |
| C | -2.686032 | -1.016123 | 0.014448 | C | -4.085504 | 0.520127 | -0.371230 |
| H | -3.715015 | -0.998718 | -0.352794 | H | -4.146640 | 0.524973 | -1.460249 |
| C | -2.640249 | -1.169173 | 1.527793 | C | -3.999216 | 1.938542 | 0.184361 |
| C | -1.935582 | 0.182811 | -0.613477 | C | -2.921827 | -0.401825 | 0.092732 |
| H | -3.127896 | -2.095466 | 1.840736 | H | -3.935696 | 1.928758 | 1.275916 |
| H | -1.611673 | -1.170339 | 1.895269 | H | -3.104272 | 2.433278 | -0.195243 |
| H | -3.191470 | -0.354531 | 2.001666 | H | -4.860720 | 2.535001 | -0.124132 |
| O | -1.610160 | 0.087069 | -1.793932 | O | -3.215151 | -1.374076 | 0.799689 |
| N | -1.639140 | 1.227126 | 0.176174 | N | -1.705649 | -0.067296 | -0.299637 |
| H | -1.817353 | 1.152579 | 1.168206 | H | -1.509576 | 0.776465 | -0.840025 |
| C | -0.555014 | 2.165652 | -0.194239 | C | -0.486631 | -0.780615 | 0.088688 |
| H | -0.665904 | 2.385327 | -1.256132 | H | -0.518628 | -0.956892 | 1.167633 |
| C | -0.626827 | 3.433222 | 0.640501 | C | -0.345385 | -2.116181 | -0.654095 |
| C | 0.726092 | 1.347868 | 0.083504 | C | 0.658656 | 0.201083 | -0.245178 |
| H | 0.166605 | 4.118918 | 0.338884 | H | -1.202192 | -2.754099 | -0.434175 |
| H | -1.587869 | 3.929957 | 0.497198 | H | -0.285314 | -1.950242 | -1.731959 |
| H | -0.482173 | 3.205281 | 1.698628 | H | 0.557195 | -2.640753 | -0.335614 |
| O | 1.165160 | 1.222641 | 1.215025 | O | 0.425584 | 1.226974 | -0.881209 |
| H | -2.481390 | -3.082856 | -0.455285 | H | -5.911767 | -0.574422 | -0.574489 |
| H | -1.986738 | -2.017185 | -1.663016 | H | -4.851079 | -1.011650 | 0.693625 |
| N | 1.227931 | 0.668837 | -0.983144 | N | 1.876260 | -0.157895 | 0.184022 |
| H | 0.696686 | 0.670132 | -1.841730 | H | 2.025857 | -1.038187 | 0.662704 |
| C | 2.240099 | -0.363517 | -0.786621 | C | 3.078073 | 0.612028 | -0.127033 |
| H | 2.309505 | -0.911036 | -1.733542 | H | 3.036800 | 0.915380 | -1.176812 |
| C | 3.618786 | 0.207039 | -0.441701 | C | 3.217127 | 1.865714 | 0.751693 |
| C | 1.715639 | -1.407000 | 0.212307 | C | 4.263019 | -0.327232 | 0.053576 |
| H | 4.360552 | -0.589060 | -0.380784 | H | 3.278135 | 1.590563 | 1.807184 |
| H | 3.916326 | 0.904826 | -1.225429 | H | 4.116290 | 2.417868 | 0.476933 |
| H | 3.585343 | 0.732860 | 0.511074 | H | 2.348396 | 2.506010 | 0.597674 |
| O | 0.551179 | -1.780923 | 0.269616 | O | 4.186313 | -1.431937 | 0.534289 |
| O | 2.665225 | -1.929296 | 0.970326 | O | 5.400980 | 0.237085 | -0.369574 |
| H | 2.282802 | -2.602491 | 1.555791 | H | 6.131673 | -0.382255 | -0.214782 |

| Isomer 3 |  |  |  | Isomer 4 |  |  |  |
| --- | --- | --- | --- | --- | --- | --- | --- |
| Atom | x | y | z | Atom | x | y | z |
| N | -1.494962 | -1.737328 | -0.775241 | N | -1.664471 | -2.531065 | -0.593240 |
| H | -1.736060 | -2.609045 | -1.248465 | H | -2.078204 | -3.457289 | -0.486819 |
| C | -2.731225 | -1.011878 | -0.249133 | C | -2.426745 | -1.453849 | 0.150546 |
| H | -3.353367 | -0.799276 | -1.121035 | H | -3.473806 | -1.554203 | -0.147206 |
| C | -3.457168 | -1.866898 | 0.771140 | C | -2.266421 | -1.628806 | 1.654102 |
| C | -2.100364 | 0.265783 | 0.348474 | C | -1.872465 | -0.155360 | -0.488564 |
| H | -3.765978 | -2.823076 | 0.341627 | H | -2.621499 | -2.611538 | 1.973082 |
| H | -2.823082 | -2.039038 | 1.642439 | H | -1.222841 | -1.520533 | 1.956644 |
| H | -4.354182 | -1.343115 | 1.105843 | H | -2.871930 | -0.893494 | 2.187092 |
| O | -1.554501 | 0.202003 | 1.430369 | O | -1.471933 | -0.219687 | -1.643225 |
| N | -2.017989 | 1.338441 | -0.494277 | N | -1.805260 | 0.957528 | 0.263825 |
| H | -2.436746 | 1.223995 | -1.405932 | H | -2.174778 | 0.970132 | 1.202787 |
| C | -0.765226 | 2.121584 | -0.497728 | C | -1.060537 | 2.132606 | -0.224377 |
| H | -0.805107 | 2.738712 | -1.399029 | H | -1.446382 | 2.401848 | -1.207964 |
| C | -0.606185 | 3.030711 | 0.718444 | C | -1.209443 | 3.300312 | 0.748486 |
| C | 0.359195 | 1.089621 | -0.773824 | C | 0.427082 | 1.783307 | -0.466141 |
| H | -1.474405 | 3.686608 | 0.793276 | H | -0.824661 | 3.045813 | 1.740762 |
| H | -0.539273 | 2.457108 | 1.642604 | H | -0.641801 | 4.152989 | 0.375244 |
| H | 0.275571 | 3.666917 | 0.604679 | H | -2.256299 | 3.599142 | 0.837147 |
| O | 0.201948 | 0.257679 | -1.678073 | O | 1.061977 | 2.346324 | -1.330479 |
| H | -0.938979 | -1.117936 | -1.408130 | H | -1.639764 | -2.243101 | -1.584369 |
| H | -0.835249 | -1.937992 | -0.005080 | H | -0.673380 | -2.532119 | -0.277599 |
| N | 1.461278 | 1.115699 | -0.013748 | N | 0.954891 | 0.838571 | 0.375418 |
| H | 1.490705 | 1.732603 | 0.784490 | H | 0.343722 | 0.390574 | 1.038744 |
| C | 2.560277 | 0.174761 | -0.184919 | C | 2.220205 | 0.186211 | 0.115769 |
| H | 2.836257 | 0.160351 | -1.244023 | H | 2.604714 | 0.589984 | -0.825075 |
| C | 3.764593 | 0.616308 | 0.655737 | C | 3.257108 | 0.425733 | 1.228641 |
| C | 2.135629 | -1.263705 | 0.138293 | C | 1.986335 | -1.305872 | -0.087933 |
| H | 4.068723 | 1.622605 | 0.362762 | H | 3.425620 | 1.498710 | 1.321978 |
| H | 3.519403 | 0.615704 | 1.721449 | H | 2.896176 | 0.044715 | 2.187817 |
| H | 4.603158 | -0.059097 | 0.496698 | H | 4.202581 | -0.061693 | 0.989257 |
| O | 1.035814 | -1.629189 | 0.489214 | O | 0.936772 | -1.889098 | 0.148505 |
| O | 3.164809 | -2.096631 | -0.042554 | O | 3.075127 | -1.925376 | -0.522651 |
| H | 2.888598 | -3.002337 | 0.168136 | H | 2.910149 | -2.878149 | -0.603911 |

| Isomer 5 |  |  |  | Isomer 6 |  |  |  |
| --- | --- | --- | --- | --- | --- | --- | --- |
| Atom | x | y | z | Atom | x | y | z |
| N | 2.676631 | -1.877640 | -0.172617 | N | 2.079259 | -1.948477 | -0.364927 |
| H | 3.081395 | -2.750522 | 0.168639 | H | 2.088309 | -1.760362 | -1.375297 |
| C | 3.559760 | -0.662736 | 0.118948 | C | 2.402668 | -0.660730 | 0.356550 |
| H | 3.823378 | -0.731264 | 1.175939 | H | 2.151449 | -0.825802 | 1.405943 |
| C | 4.785227 | -0.652681 | -0.773862 | C | 3.875337 | -0.298634 | 0.178719 |
| C | 2.555065 | 0.474030 | -0.170060 | C | 1.459782 | 0.386111 | -0.279282 |
| H | 5.417852 | 0.195830 | -0.507212 | H | 4.525407 | -1.072446 | 0.594086 |
| H | 5.376161 | -1.563805 | -0.652885 | H | 4.115831 | -0.154514 | -0.877495 |
| H | 4.491806 | -0.538122 | -1.819436 | H | 4.091190 | 0.633187 | 0.703499 |
| O | 2.196931 | 0.646414 | -1.319116 | O | 1.074450 | 0.198989 | -1.426851 |
| N | 1.997459 | 1.062223 | 0.921360 | N | 1.118453 | 1.435148 | 0.499906 |
| H | 2.338987 | 0.755801 | 1.820191 | H | 1.406905 | 1.400054 | 1.468593 |
| C | 0.585608 | 1.454916 | 0.895293 | C | -0.055866 | 2.319422 | 0.239377 |
| H | 0.335885 | 1.728192 | 1.924859 | H | -0.044718 | 3.055423 | 1.042589 |
| C | 0.303148 | 2.660017 | -0.010809 | C | -0.024539 | 3.036062 | -1.105725 |
| C | -0.257571 | 0.183942 | 0.578688 | C | -1.247836 | 1.393976 | 0.551583 |
| H | 0.992677 | 3.466025 | 0.243150 | H | -0.018192 | 2.353798 | -1.953536 |
| H | 0.435491 | 2.403012 | -1.059893 | H | -0.895599 | 3.690215 | -1.179444 |
| H | -0.709745 | 3.037626 | 0.144733 | H | 0.872451 | 3.654709 | -1.168390 |
| O | 0.243017 | -0.955561 | 0.596535 | O | -1.631229 | 1.270405 | 1.713970 |
| H | 1.718486 | -1.701459 | 0.242327 | H | 2.747412 | -2.687428 | -0.142126 |
| H | 2.540824 | -1.948790 | -1.185708 | H | 1.097447 | -2.273574 | -0.152099 |
| N | -1.552539 | 0.382665 | 0.344589 | N | -1.683263 | 0.589568 | -0.449792 |
| H | -1.931542 | 1.320124 | 0.273803 | H | -1.193941 | 0.630762 | -1.335116 |
| C | -2.496767 | -0.697454 | 0.060908 | C | -2.363311 | -0.686938 | -0.152010 |
| H | -2.037407 | -1.385339 | -0.653353 | H | -3.130870 | -0.488977 | 0.597910 |
| C | -2.900809 | -1.470673 | 1.326194 | C | -2.966594 | -1.292071 | -1.407939 |
| C | -3.708088 | -0.052023 | -0.602676 | C | -1.253076 | -1.563217 | 0.461409 |
| H | -3.382952 | -0.805271 | 2.045783 | H | -3.461160 | -2.234898 | -1.170799 |
| H | -3.593507 | -2.272035 | 1.067871 | H | -3.704578 | -0.607390 | -1.828330 |
| H | -2.011332 | -1.905049 | 1.782843 | H | -2.194885 | -1.495981 | -2.152731 |
| O | -3.883823 | 1.139398 | -0.671194 | O | -0.597500 | -2.358643 | -0.187880 |
| O | -4.552257 | -0.976440 | -1.073551 | O | -0.921554 | -1.314064 | 1.726166 |
| H | -5.321690 | -0.530207 | -1.462129 | H | -1.406322 | -0.527830 | 2.067099 |

| Isomer 7 |  |  |  | Isomer 8 |  |  |  |
| --- | --- | --- | --- | --- | --- | --- | --- |
| Atom | x | y | z | Atom | x | y | z |
| N | -5.300623 | -0.221115 | 0.131930 | N | 4.347076 | -0.969727 | -0.252160 |
| H | -5.871061 | 0.362962 | 0.744750 | H | 4.915906 | -1.031694 | -1.096439 |
| C | -4.077329 | 0.500795 | -0.400986 | C | 2.855762 | -1.068916 | -0.516008 |
| H | -4.127428 | 0.483336 | -1.490451 | H | 2.714871 | -1.235877 | -1.584622 |
| C | -4.004247 | 1.930719 | 0.126462 | C | 2.218912 | -2.183944 | 0.308863 |
| C | -2.913432 | -0.404929 | 0.092928 | C | 2.352713 | 0.347741 | -0.119158 |
| H | -4.865911 | 2.515814 | -0.202638 | H | 2.649960 | -3.154163 | 0.050803 |
| H | -3.951551 | 1.943546 | 1.218559 | H | 2.347703 | -1.999365 | 1.378933 |
| H | -3.108464 | 2.422905 | -0.254492 | H | 1.147986 | -2.229064 | 0.105726 |
| O | -3.206938 | -1.364313 | 0.816815 | O | 3.177284 | 1.128984 | 0.364933 |
| N | -1.695392 | -0.070822 | -0.295167 | N | 1.065458 | 0.597855 | -0.320070 |
| H | -1.499514 | 0.763282 | -0.850391 | H | 0.454192 | -0.152027 | -0.642169 |
| C | -0.475739 | -0.767674 | 0.119246 | C | 0.385592 | 1.795643 | 0.230444 |
| H | -0.518475 | -0.923295 | 1.201200 | H | 0.978438 | 2.142311 | 1.076082 |
| C | -0.317804 | -2.116119 | -0.596275 | C | 0.249177 | 2.905031 | -0.809509 |
| C | 0.666056 | 0.215982 | -0.222323 | C | -0.906961 | 1.196585 | 0.814964 |
| H | -1.171891 | -2.756309 | -0.372399 | H | 1.239021 | 3.270488 | -1.087776 |
| H | -0.248242 | -1.970243 | -1.676403 | H | -0.242540 | 2.560269 | -1.723641 |
| H | 0.585955 | -2.627293 | -0.259760 | H | -0.320590 | 3.740688 | -0.397937 |
| O | 0.429993 | 1.227245 | -0.878638 | O | -0.914955 | 0.713840 | 1.930121 |
| H | -5.896114 | -0.606767 | -0.601105 | H | 4.419824 | 0.009490 | 0.164959 |
| H | -4.849150 | -1.012837 | 0.687264 | H | 4.675581 | -1.665691 | 0.418376 |
| N | 1.886478 | -0.127815 | 0.218528 | N | -1.960802 | 1.091213 | -0.050764 |
| H | 2.014871 | -0.971676 | 0.758261 | H | -1.847398 | 1.440855 | -0.990789 |
| C | 3.073753 | 0.677888 | -0.080815 | C | -2.930173 | 0.026715 | 0.132909 |
| H | 2.950253 | 1.087635 | -1.084786 | H | -3.253954 | 0.027284 | 1.175664 |
| C | 3.253931 | 1.838422 | 0.912161 | C | -4.139873 | 0.208431 | -0.789938 |
| C | 4.336640 | -0.172906 | -0.109204 | C | -2.242845 | -1.324834 | -0.109320 |
| H | 3.373506 | 1.462160 | 1.930977 | H | -4.624712 | 1.160682 | -0.571221 |
| H | 4.136360 | 2.420721 | 0.644187 | H | -3.839241 | 0.200809 | -1.842018 |
| H | 2.376867 | 2.485250 | 0.870153 | H | -4.858699 | -0.595141 | -0.633735 |
| O | 5.376356 | 0.206925 | -0.568069 | O | -1.115710 | -1.471482 | -0.542501 |
| O | 4.171105 | -1.382967 | 0.484667 | O | -3.044502 | -2.341320 | 0.206006 |
| H | 5.021646 | -1.849116 | 0.464675 | H | -2.589236 | -3.180135 | 0.032798 |

| Isomer 9 |  |  |  | Isomer 10 |  |  |  |
| --- | --- | --- | --- | --- | --- | --- | --- |
| Atom | x | y | z | Atom | x | y | z |
| N | 1.984979 | -2.110832 | -0.401106 | N | -5.303085 | -0.220585 | 0.155824 |
| H | 2.461104 | -3.004112 | -0.272407 | H | -5.871729 | 0.343798 | 0.788408 |
| C | 2.497187 | -1.033138 | 0.531507 | C | -4.086619 | 0.520481 | -0.365945 |
| H | 2.093873 | -1.260339 | 1.519148 | H | -4.146940 | 0.537130 | -1.454895 |
| C | 4.020653 | -1.000803 | 0.527639 | C | -4.011377 | 1.933179 | 0.205753 |
| C | 1.878858 | 0.247129 | -0.082668 | C | -2.916497 | -0.397432 | 0.089348 |
| H | 4.370042 | -0.209662 | 1.193148 | H | -4.876927 | 2.526723 | -0.096970 |
| H | 4.440905 | -1.945274 | 0.882626 | H | -3.948952 | 1.911554 | 1.297187 |
| H | 4.398497 | -0.788671 | -0.475188 | H | -3.119751 | 2.438674 | -0.167402 |
| O | 1.855362 | 0.323544 | -1.299037 | O | -3.202020 | -1.376660 | 0.789048 |
| N | 1.388620 | 1.157297 | 0.786890 | N | -1.701816 | -0.052855 | -0.301322 |
| H | 1.415310 | 0.902090 | 1.763091 | H | -1.512564 | 0.793925 | -0.838120 |
| C | 0.356243 | 2.162215 | 0.412523 | C | -0.481567 | -0.768616 | 0.079276 |
| H | 0.143861 | 2.709264 | 1.335045 | H | -0.513437 | -0.956375 | 1.156248 |
| C | 0.799848 | 3.133499 | -0.670982 | C | -0.338978 | -2.096861 | -0.676971 |
| C | -0.882670 | 1.330914 | -0.001834 | C | 0.664751 | 0.213580 | -0.243676 |
| H | 1.731133 | 3.621216 | -0.378305 | H | -0.279058 | -1.920316 | -1.753174 |
| H | 0.938300 | 2.615165 | -1.616626 | H | 0.563532 | -2.624422 | -0.363354 |
| H | 0.031269 | 3.897666 | -0.802367 | H | -1.195567 | -2.737067 | -0.463291 |
| O | -1.150515 | 1.087548 | -1.160974 | O | 0.439087 | 1.244700 | -0.874008 |
| H | 0.956785 | -2.217323 | -0.273571 | H | -5.902046 | -0.589212 | -0.583134 |
| H | 2.131426 | -1.755778 | -1.359139 | H | -4.843644 | -1.025528 | 0.685816 |
| N | -1.574161 | 0.766301 | 1.035983 | N | 1.880007 | -0.151928 | 0.188561 |
| H | -1.332318 | 0.988136 | 1.988129 | H | 2.034270 | -1.041097 | 0.651430 |
| C | -2.546778 | -0.290465 | 0.789935 | C | 3.087937 | 0.600504 | -0.128707 |
| H | -2.787436 | -0.726956 | 1.766446 | H | 3.039950 | 0.896095 | -1.182665 |
| C | -3.842088 | 0.210552 | 0.140695 | C | 3.230673 | 1.858465 | 0.745268 |
| C | -1.859436 | -1.436968 | 0.032738 | C | 4.265640 | -0.364695 | 0.067405 |
| H | -3.638281 | 0.618640 | -0.847748 | H | 3.316527 | 1.585077 | 1.798825 |
| H | -4.565418 | -0.599488 | 0.049955 | H | 4.111943 | 2.441549 | 0.467089 |
| H | -4.269995 | 0.993383 | 0.768514 | H | 2.353526 | 2.490331 | 0.605655 |
| O | -0.682135 | -1.733902 | 0.169784 | O | 4.135290 | -1.453618 | 0.558550 |
| O | -2.699121 | -2.127100 | -0.725017 | O | 5.471649 | 0.079652 | -0.319914 |
| H | -2.234764 | -2.865644 | -1.148495 | H | 5.429299 | 0.962524 | -0.709001 |

| Isomer 11 |  |  |  | Isomer 12 |  |  |  |
| --- | --- | --- | --- | --- | --- | --- | --- |
| Atom | x | y | z | Atom | x | y | z |
| N | 5.204408 | 0.069305 | -0.160004 | N | -1.581630 | -2.243531 | 0.285305 |
| H | 5.717554 | 0.104081 | -1.041407 | H | -1.917711 | -3.192930 | 0.114670 |
| C | 3.910219 | -0.718467 | -0.247012 | C | -2.410282 | -1.190363 | -0.433451 |
| H | 3.838997 | -1.132282 | -1.253794 | H | -2.222429 | -1.323359 | -1.500051 |
| C | 3.862069 | -1.819883 | 0.808342 | C | -3.885326 | -1.349220 | -0.089706 |
| C | 2.830968 | 0.382260 | -0.045810 | C | -1.802456 | 0.118456 | 0.114660 |
| H | 2.918211 | -2.362119 | 0.738408 | H | -4.047810 | -1.220334 | 0.982814 |
| H | 4.667252 | -2.542623 | 0.658779 | H | -4.461917 | -0.584507 | -0.612822 |
| H | 3.931531 | -1.401169 | 1.816005 | H | -4.265305 | -2.325051 | -0.402172 |
| O | 3.220481 | 1.524378 | 0.218501 | O | -1.540208 | 0.141538 | 1.315090 |
| N | 1.566878 | 0.006275 | -0.164461 | N | -1.546804 | 1.091260 | -0.770733 |
| H | 1.296296 | -0.959027 | -0.344486 | H | -1.723744 | 0.888520 | -1.745003 |
| C | 0.428106 | 0.906001 | 0.036057 | C | -0.581542 | 2.199908 | -0.529453 |
| H | 0.601443 | 1.479974 | 0.950655 | H | -0.608308 | 2.804250 | -1.435325 |
| C | 0.241266 | 1.860576 | -1.153263 | C | -0.919605 | 3.077083 | 0.672785 |
| C | -0.786901 | -0.019653 | 0.220588 | C | 0.812930 | 1.530150 | -0.545256 |
| H | 0.032590 | 1.299749 | -2.066683 | H | -1.908903 | 3.519608 | 0.541206 |
| H | -0.588710 | 2.546517 | -0.974271 | H | -0.915924 | 2.528243 | 1.612336 |
| H | 1.144724 | 2.455343 | -1.290711 | H | -0.189243 | 3.886370 | 0.733106 |
| O | -0.683224 | -1.228681 | -0.017090 | O | 1.491019 | 1.520553 | -1.556665 |
| H | 4.849058 | 1.041380 | 0.086648 | H | -0.581419 | -2.155490 | 0.011466 |
| H | 5.830330 | -0.274891 | 0.569624 | H | -1.630719 | -2.024734 | 1.290326 |
| N | -1.931176 | 0.554944 | 0.611275 | N | 1.172951 | 0.858765 | 0.583843 |
| H | -1.969948 | 1.556908 | 0.736426 | H | 0.533729 | 0.813821 | 1.364169 |
| C | -3.186895 | -0.196245 | 0.817424 | C | 2.357049 | 0.020715 | 0.597368 |
| H | -2.911408 | -1.144244 | 1.288615 | H | 3.226280 | 0.617594 | 0.313701 |
| C | -4.123899 | 0.595484 | 1.716403 | C | 2.575342 | -0.580111 | 1.991479 |
| C | -3.835345 | -0.506165 | -0.557691 | C | 2.330084 | -1.094963 | -0.445989 |
| H | -3.651012 | 0.794620 | 2.680471 | H | 1.724502 | -1.195858 | 2.297952 |
| H | -4.413452 | 1.536522 | 1.243559 | H | 3.473212 | -1.198310 | 1.999147 |
| H | -5.038506 | 0.027610 | 1.885159 | H | 2.703306 | 0.222138 | 2.720329 |
| O | -4.859645 | -0.003339 | -0.919832 | O | 3.293087 | -1.559178 | -0.969591 |
| O | -3.157612 | -1.377247 | -1.327482 | O | 1.057392 | -1.620852 | -0.729084 |
| H | -2.333250 | -1.664345 | -0.893666 | H | 1.233321 | -2.251842 | -1.447376 |

| Isomer 13 |  |  |  | Isomer 14 |  |  |  |
| --- | --- | --- | --- | --- | --- | --- | --- |
| Atom | x | y | z | Atom | x | y | z |
| N | -1.582425 | -2.243367 | 0.284772 | N | -0.860257 | -2.474442 | 0.582374 |
| H | -0.582140 | -2.155640 | 0.010839 | H | -1.007427 | -3.480499 | 0.679013 |
| C | -2.410672 | -1.189784 | -0.433780 | C | -1.919013 | -1.806460 | -0.280290 |
| H | -2.223018 | -1.322886 | -1.500377 | H | -1.685897 | -2.054246 | -1.317068 |
| C | -3.885744 | -1.347980 | -0.089967 | C | -3.307831 | -2.286414 | 0.123022 |
| C | -1.802172 | 0.118729 | 0.114242 | C | -1.690441 | -0.312264 | 0.042355 |
| H | -4.462096 | -0.583057 | -0.613013 | H | -4.057581 | -1.782927 | -0.489722 |
| H | -4.266129 | -2.323657 | -0.402430 | H | -3.419341 | -3.362405 | -0.032255 |
| H | -4.048118 | -1.219095 | 0.982562 | H | -3.510499 | -2.044834 | 1.168927 |
| O | -1.539580 | 0.141439 | 1.314730 | O | -1.423237 | -0.029011 | 1.202279 |
| N | -1.545804 | 1.091307 | -0.770927 | N | -1.693639 | 0.556049 | -0.983417 |
| H | -1.723817 | 0.889394 | -1.745173 | H | -1.981332 | 0.242960 | -1.899239 |
| C | -0.580779 | 2.200095 | -0.529271 | C | -1.184265 | 1.938013 | -0.825662 |
| H | -0.607219 | 2.804291 | -1.435204 | H | -1.082010 | 2.329324 | -1.842246 |
| C | -0.919363 | 3.077461 | 0.672692 | C | -2.120871 | 2.836673 | -0.022851 |
| C | 0.813699 | 1.530120 | -0.544571 | C | 0.245159 | 1.907729 | -0.209281 |
| H | -1.908725 | 3.519746 | 0.540781 | H | -3.091312 | 2.902803 | -0.518102 |
| H | -0.915763 | 2.528918 | 1.612385 | H | -2.251070 | 2.447818 | 0.986561 |
| H | -0.189246 | 3.886976 | 0.732975 | H | -1.688419 | 3.833930 | 0.049326 |
| O | 1.492247 | 1.520332 | -1.555637 | O | 0.646377 | 2.805939 | 0.495639 |
| H | -1.631274 | -2.024480 | 1.289806 | H | 0.093785 | -2.287766 | 0.217188 |
| H | -1.918926 | -3.192588 | 0.114153 | H | -0.919590 | -2.010305 | 1.504403 |
| N | 1.173262 | 0.858776 | 0.584741 | N | 0.987901 | 0.803093 | -0.530360 |
| H | 0.532989 | 0.812561 | 1.364108 | H | 0.648095 | 0.187543 | -1.249547 |
| C | 2.356884 | 0.020037 | 0.598020 | C | 2.278983 | 0.495699 | 0.079224 |
| H | 3.226508 | 0.616700 | 0.315235 | H | 3.030473 | 1.230981 | -0.217509 |
| C | 2.574247 | -0.582241 | 1.991622 | C | 2.214736 | 0.437312 | 1.620299 |
| C | 2.329703 | -1.094683 | -0.446252 | C | 2.783772 | -0.842319 | -0.423086 |
| H | 1.722916 | -1.197785 | 2.297160 | H | 1.462324 | -0.283027 | 1.949489 |
| H | 3.471741 | -1.200994 | 1.999252 | H | 3.189949 | 0.154882 | 2.020195 |
| H | 2.702342 | 0.219215 | 2.721307 | H | 1.943452 | 1.418057 | 2.005049 |
| O | 3.292411 | -1.557696 | -0.971367 | O | 3.919699 | -1.135202 | -0.623078 |
| O | 1.057008 | -1.621863 | -0.728278 | O | 1.765067 | -1.798776 | -0.597571 |
| H | 1.233243 | -2.252847 | -1.446468 | H | 2.223740 | -2.586598 | -0.935166 |
